# Loss of Vascular TMEM16A Impairs Cerebral Autoregulation and Exacerbates Ischemia–reperfusion Injury

**DOI:** 10.1007/s12975-026-01471-4

**Published:** 2026-07-22

**Authors:** Ask Carit Andersen, Ida Damsgaard Larsen, Elizaveta V. Melnikova, Marlon Gernemann, Boris V. Skryabin, Tina Myhre Pedersen, Hans Christian Beck, Halvor Østerby Guldbrandsen, Eugenio Gutierrez, Christian Aalkjaer, Dmitry D. Postnov, Vladimir V. Matchkov, Line Mathilde Brostrup Hansen

**Affiliations:** 1 Department of Biomedicine, Health, Aarhus University, Aarhus, Denmark; 2 Medical Faculty, Core Facility Transgenic Animal and Genetic Engineering Models (TRAM), University of Muenster, Muenster, Germany; 3 Department of Clinical Research, University of Southern Denmark, Odense, Denmark; 4 Department of Clinical Medicine, Aarhus University, Aarhus, Denmark; 5 Center of Functionally Integrative Neuroscience, Department of Clinical Medicine, Aarhus University, Aarhus, Denmark

**Keywords:** Ischemic stroke, Myogenic tone, TMEM16A, Reperfusion, Cerebral blood flow autoregulation

## Abstract

TMEM16A forms a Ca²⁺-activated Cl⁻ channel in vascular mural cells (smooth muscle cells and pericytes) that generates depolarizing Cl⁻ efflux upon intracellular Ca²⁺ elevation, thereby amplifying agonist-induced vasoconstriction. TMEM16A has been implicated in excessive capillary pericyte constriction following cerebral ischemia, suggesting that its inhibition may improve post-stroke recovery. However, the impact of systemic vascular TMEM16A inhibition on focal reperfusion efficiency and cerebrovascular autoregulation remains unknown. To address this question, mice with inducible mural cell-specific (Myosin Heavy Chain 11 promoter controlled) deletion of TMEM16A were subjected to transient middle cerebral artery occlusion. Reperfusion dynamics and stroke-reperfusion outcome were assessed using laser speckle contrast imaging, cylinder test for motor function, and infarct quantification by 2,3,5-triphenyltetrazolium chloride staining. Systemic cardiovascular parameters were monitored with radiotelemetry. Middle cerebral artery myogenic tone was assessed with pressure myography. Mice lacking TMEM16A in mural cells exhibited impaired reperfusion and worsened stroke outcome compared with wild-type controls, despite unchanged systemic cardiovascular parameters. In wild-type mice, capillary pericytes maintained basal contractile tone in both hemispheres, and this was further enhanced in peri-infarct cortex. In contrast, TMEM16A-deficient capillary pericytes lacked basal tone in both the ipsilateral and contralateral hemispheres. TMEM16A-deficient middle cerebral arteries failed to develop pressure-induced myogenic tone. These findings demonstrate that TMEM16A is required for effective cerebral autoregulation and that its deficiency significantly impairs post-ischemic reperfusion. The results caution against systemic TMEM16A inhibition as a therapeutic strategy for stroke and highlight the need for spatially restricted approaches to modulate cerebral perfusion via the Ca²⁺-activated Cl⁻ channels.

**Graphical Abstract:**

**Figure:**
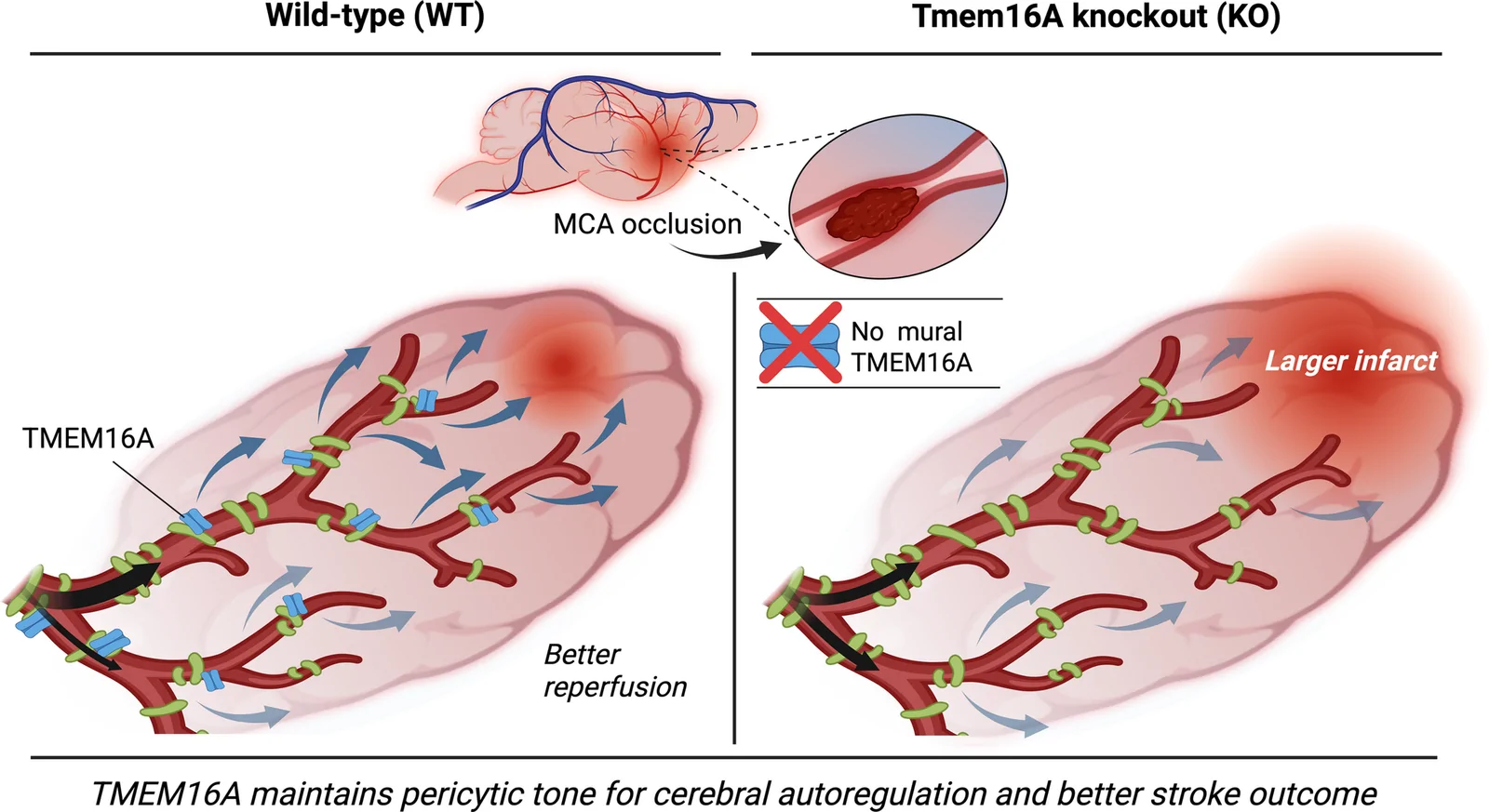

**Supplementary Information:**

The online version contains supplementary material available at https://doi.org/10.1007/s12975-026-01471-4.

## Introduction

Vascular smooth muscle cells and pericytes, collectively referred to as mural cells, actively accumulate intracellular Cl⁻, resulting in an equilibrium potential that is more positive than the resting membrane potential [1]. Consequently, activation of Cl⁻ conductance in these cells produces membrane depolarization. Mural cells express TMEM16A, a Ca²⁺-activated Cl⁻ channel that depolarizes the plasma membrane in response to elevations in intracellular Ca²⁺ [2–16]. Owing to these properties, TMEM16A functions as an efficient amplifier of agonist-induced [4, 8, 9, 13] and myogenic contraction [3], in which an initial rise in intracellular Ca²⁺ promotes further depolarization via TMEM16A channels, enhances voltage-gated Ca²⁺ influx, and strengthens contractile responses [17–19].

The TMEM16A expression in capillary pericytes [4, 13] and cerebrovascular smooth muscle cells [3, 20] positions this channel as a key regulator of cerebral circulation, where changes in cerebrovascular resistance redistribute local blood flow in accordance with metabolic demand [21–23]. In line with this concept, TMEM16A has been implicated in ischemia–reperfusion injury. In experimental models of ischemic stroke, TMEM16A was shown to amplify agonist-induced pericyte contraction, leading to impaired reperfusion and increased infarct size [13]. Accordingly, inhibition of TMEM16A has been proposed as a therapeutic strategy to improve post-ischemic cerebral perfusion [15, 19]. However, this proposal has largely been based on studies using focal application of putative TMEM16A inhibitors, many of which display limited specificity in complex tissue systems [5]. Interpretation is further complicated by the widespread expression of TMEM16A in the brain, including epithelial cells, glia, cerebrovascular smooth muscle and endothelial cells, pericytes, and neurons [24, 25], making it difficult to distinguish vascular from non-vascular effects.

This consideration is essential for evaluating any therapeutic strategy involving systemic TMEM16A inhibition. Beyond its role in pericyte-mediated capillary constriction following ischemia [13], TMEM16A has been reported to be upregulated under hypoxic conditions, where it promotes proliferation and migration of brain capillary endothelial cells [26]. However, this observation was not supported by our previous spatial transcriptomics analysis of ischemic stroke mice, which revealed a modest bulk reduction in TMEM16A mRNA in the ipsilateral hemisphere compared with the contralateral hemisphere [27]. Pharmacological inhibition of TMEM16A has also been reported to preserve blood–brain barrier integrity after stroke [28]. In addition, TMEM16A expression has been linked to cerebrovascular remodeling and smooth muscle cell proliferation [29].

Collectively, these findings indicate that TMEM16A signaling extends beyond its function as a Ca²⁺-activated Cl⁻ channel, and in addition to ion conductance, TMEM16A has been shown to modulate intracellular signaling pathways and gene expression [24]. Specifically, TMEM16A can activate epidermal growth factor receptors and trigger phosphorylation of downstream kinases, including ERK1/2, phospholipase Cγ, AKT, and Ca²⁺/calmodulin-dependent kinase II. Although these mechanisms have been primarily described in cancer cells [30, 31], the same pathways are highly relevant for mural cell function. TMEM16A has also been reported to regulate protein expression via modulation of STAT transcription factors, potentially downstream of epidermal growth factor receptor signaling [32]. Together, these non-canonical functions of TMEM16A may contribute to functional and phenotypic changes in mural cells and their neighboring cell types following ischemic stroke and reperfusion, forming complex intercellular signaling networks within the neurovascular unit.

The present study investigates the role of TMEM16A expression in cerebral mural cells in determining ischemic stroke–reperfusion outcome using a mouse model with mural-cell-specific deletion of TMEM16A. Transient focal cerebral ischemia was induced by occlusion of a branch of the middle cerebral artery (MCA), and the consequences of TMEM16A ablation were evaluated in terms of reperfusion dynamics, infarct size, and behavioral outcomes. These functional assessments were complemented by analyses of systemic cardiovascular parameters, cerebrovascular myogenic tone, and cortical proteomic profiles in peri-infarct and control regions from mural-cell-specific TMEM16A knockout (TMEM16A SM-KO) and wild-type (WT) mice. The findings demonstrate that loss of TMEM16A leads to impaired regulatory capacity of cerebral microcirculation, ultimately compromising efficient reperfusion following transient cerebral ischemia.

## Materials and Methods

Detailed Methods are available as *Supplementary*.

### Animals and Ethical Approval

All animal experiments were conducted in accordance with the European Communities Council Directive (86/609/EEC) for the Protection of Animals used for Experimental and other Scientific Purposes and approved by the Animal Experiments Inspectorate of the Danish Ministry of Environment and Food. Male mice (2–5 months) were utilized throughout and housed under standard conditions (12 h light/dark cycle, 21 °C, ad libitum access to food and water).

### Generation of Smooth Muscle Cell-specific TMEM16A Knockout Mice

Smooth muscle cell-specific TMEM16A knockout mice were generated using a tamoxifen-inducible Cre-loxP system [33]. Mice carrying floxed TMEM16A alleles were crossed with smooth muscle myosin heavy chain (Myh11)-SMMHC-Cre^ERT2^ mice, in which Cre recombinase is expressed under control of the Myh11 promoter [34]. Gene deletion was induced by tamoxifen injection (20 mg•kg^− 1^, i.p.) for 5 consecutive days, and experiments were performed at least a week later. SMMHC-Cre^ERT2^ mice without floxed alleles served as controls. Knockdown efficiency was confirmed by reduced TMEM16A protein expression in vascular tissue, as expressed by Western blotting and immunohistochemistry.

### Voltage Clamp Recordings of Ca^2+^-activated Cl^-^ current in Smooth Muscle Cells

To assess TMEM16A-dependent Ca^2+^-activated Cl^−^ current, smooth muscle cells were isolated from middle cerebral arteries and studied utilizing whole-cell patch-clamp recordings. Current-voltage relationships were obtained using a voltage-step protocol (− 60 to + 60 mV) with 900 nM of free Ca^2+^ in the patch pipette as it has previously been shown to induce maximal activation of conventional Ca^2+^-activated Cl^−^ current in smooth muscle cells [35]. TMEM16A-specific current was pharmacologically inhibited using the selective inhibitor of the TMEM16A-dependent Ca^2+^-activated Cl^−^ channel, Ani9 (10 µM) [36].

### Temporal Transient Middle Cerebral Artery Occlusion and In Vivo Blood Flow Index Measurements

Focal cerebral ischemia was induced by transient middle cerebral artery occlusion. Under isoflurane anesthesia, the distal middle cerebral artery was mechanically occluded for 1 h, followed by 24 h of reperfusion. Cortical perfusion was assessed in vivo using laser speckle contrast imaging at baseline, during occlusion, immediately after reperfusion, and after 24 h. Blood flow index (BFI) values were calculated from Laser Speckle Contrast Imaging [37–39] and analyzed within defirent size cerebral blood vessels and the parenchyma. Mice exhibiting parenchymal BFI reduction greater than 50% were included.

### Behavior Assessment

Sensorimotor function was evaluated using the cylinder test at baseline and 24 h after ischemia-reperfusion. Forelimb use asymmetry and locomotor activity were quantified from blinded video recordings.

### Infarct Validation and Edema Development

Brain infarct volume was assessed with 2,3,5-triphenyltetrazolium chloride (TTC) staining of coronal brain sections, and infarct size was quantified as the percentage of total hemispheric volume. Edema changes in the ipsilateral hemisphere were assessed in relation to the contralateral hemisphere volume.

### Histology and Immunofluorescence

Pericytes and smaller vascular structures were visualized in brain sections using immunofluorescent labeling. Microvascular density and lacunarity were quantified using lectin labeling. Association of lectin-labeled capillaries with pericytes was based on colocalization of PDGFRβ, and capillary diameter was measured near adjacent pericyte soma and without it. For TMEM16A mural cell localization and knockout efficiency, coronal brain slices were stained for TMEM16A and α-smooth-muscle-actin.

### Proteomics

Cortical tissue samples were analyzed using tandem mass tag-based quantitative proteomics followed by reversed-phase nano-liquid chromatography tandem mass spectrometry. Protein identification and quantification were performed using established database search algorithms (Uniprot mouse database downloaded on 30th September 2019).

### Pressure Myography

Middle cerebral arterial segments were dissected in ice-cold physiological salt solution (PSS) and cannulated from both ends with glass microcannulas in a pressure myograph (111P, DMT) [37, 40]. The pressure steps between 40 and 120 mmHg with 20 mmHg increments, 5 min each, were repeated under control conditions in PSS and after 15 min incubation in Ca^2+^-free PSS in the presence of 10 µM nifedipine and 10 µM Y27632. The outer diameters were measured, and the degree of tone at each pressure step was quantified.

### Radiotelemetry

In a subset of animals, blood pressure, heart rate, and activity were continuously recorded using implanted radiotelemetry probes (Data Sciences International) in freely moving mice.

### Data Analysis and Statistics

Experiments were randomized and analyzed in a blind manner, with genotypes being blinded. Data were compared using unpaired or paired *t*-tests, one-way or two-way ANOVA, followed by multiple-comparisons correction, where appropriate. The statistical test used is specifically indicated in the figure legends. All data are represented as mean ± Standard Deviation (SD). An asterisk (*) indicates analysis within the WT group, an octothorpe (#) indicates the intervention effect within the TMEM16A SM-KO group, whereas a double dagger (‡) indicates the comparisons between WT and TMEM16A SM-KO at the same condition or time point. *P*-values below 0.05 are considered significant, with the number of symbols indicating the degree of significance.

## Results

**Fig. 1:**
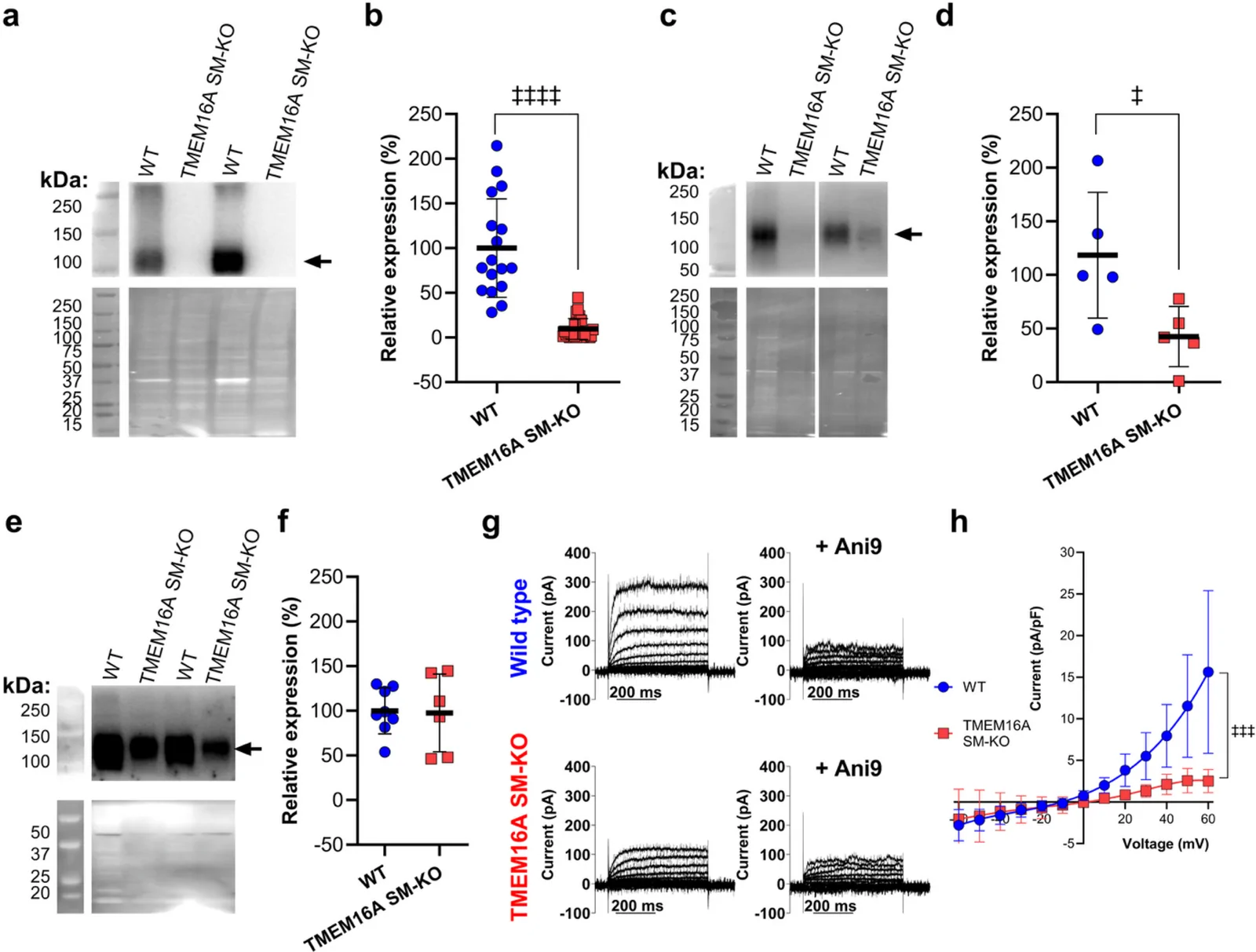
TMEM16A knockout in vascular smooth muscle cells. Tamoxifen treatment induced smooth muscle cell-specific TMEM16A knockout (TMEM16A SM-KO) in mice carrying floxed TMEM16A alleles and Cre recombinase under control of SMMHC promoter, but not in mice expressing Cre alone (WT). This was demonstrated by Western blot analysis of aortic lysates, in which TMEM16A was detected in WT and not in TMEM16A SM-KO samples. Representative blots in (**a**) with an arrow indicating the band corresponding to TMEM16A, and averaged densitometric analysis in (**b**). Similarly, lysates of MCA suppressed expression TMEM16A in TMEM16A SM-KO mice in comparison with WT; representative blots in (**c**) and averaged densitometric analysis in (**d**). In contrast, TMEM16A expression in the lysates from left ventricles of the heart did not differ between WT and TMEM16A SM-KO mice; representative blots in (**e**) with an arrow indicating the band corresponding to TMEM16A, and averaged densitometric analysis in (**f**). Upper panels in the representative images in (**a**), (**c**), and (**e**) show membranes stained for TMEM16A with corresponding molecular weight markers. The lower panels in (**a**) and (**c**) show total protein loading assessed using a stain-free gel with the corresponding molecular weight range. The lower panel in (**e**) shows loading control using staining for pan-actin with corresponding markers. ‡ and ‡‡‡‡, *P* < 0.05 and < 0.0001; compared with Welch’s *t* test for *n* = 17–28 (in b), *n =* 5 (in (**d**) and *n =* 6–8 (in **f**). Whole-cell patch clamp demonstrated an Ani9-sensitive current in middle cerebral artery smooth muscle cells from WT, yet not from TMEM16A SM-KO mice. Membrane currents prior to, and after incubation with 10 µM Ani9 were measured at 500 ms voltage steps between − 60 and + 60 mV with increments of 10 mV, as shown in representative traces for smooth muscle cells isolated from middle cerebral artery of WT and TMEM16A SM-KO mice (**g**). Averaged Ani9-sensitive membrane current (**h**) was normalized to cell capacitance (12.3 ± 1.2 pF for WT and 12.0 ± 0.8 pF for TMEM16A SM-KO). Current–voltage relationships were fitted with a fifth-order polynomial and compared between genotypes using an extra-sum-of-squares *F*-test. The current in TMEM16A SM-KO smooth muscle cells was not significantly different from zero. ‡‡‡ and ‡‡‡‡, *P* < 0.001 and < 0.0001. *n* = 5–6

Tamoxifen injections successfully induced TMEM16A knockout in smooth muscle cells of SMMHC-CreER^T2^ mice carrying floxed TMEM16A (TMEM16A SM-KO), whereas TMEM16A expression was preserved in SMMHC-CreER^T2^ mice used as WT controls. Loss of TMEM16A expression in smooth muscle cells was confirmed by Western blot analysis. Both aorta (Fig. 1a, b) and MCA (Fig. 1c, d) showed diminished TMEM16A in the knockout group. In contrast, no difference in TMEM16A expression in cardiac tissue was observed between TMEM16A SM-KO and WT groups (Fig. 1e, f). Functional deletion of TMEM16A was further confirmed by the voltage-clamp experiments. In smooth muscle cells isolated from the MCAs of WT mice, intracellular Ca^2+^ (~ 900 nM) evoked an outward rectifying current [35] that was sensitive to 10 µM Ani9, an inhibitor of TMEM16A-dependent Ca^2+^-activated Cl^−^ current [36]. A similar current was negligible in smooth muscle cells from TMEM16A SM-KO mice (Fig. 1g, h).

**Fig. 2:**
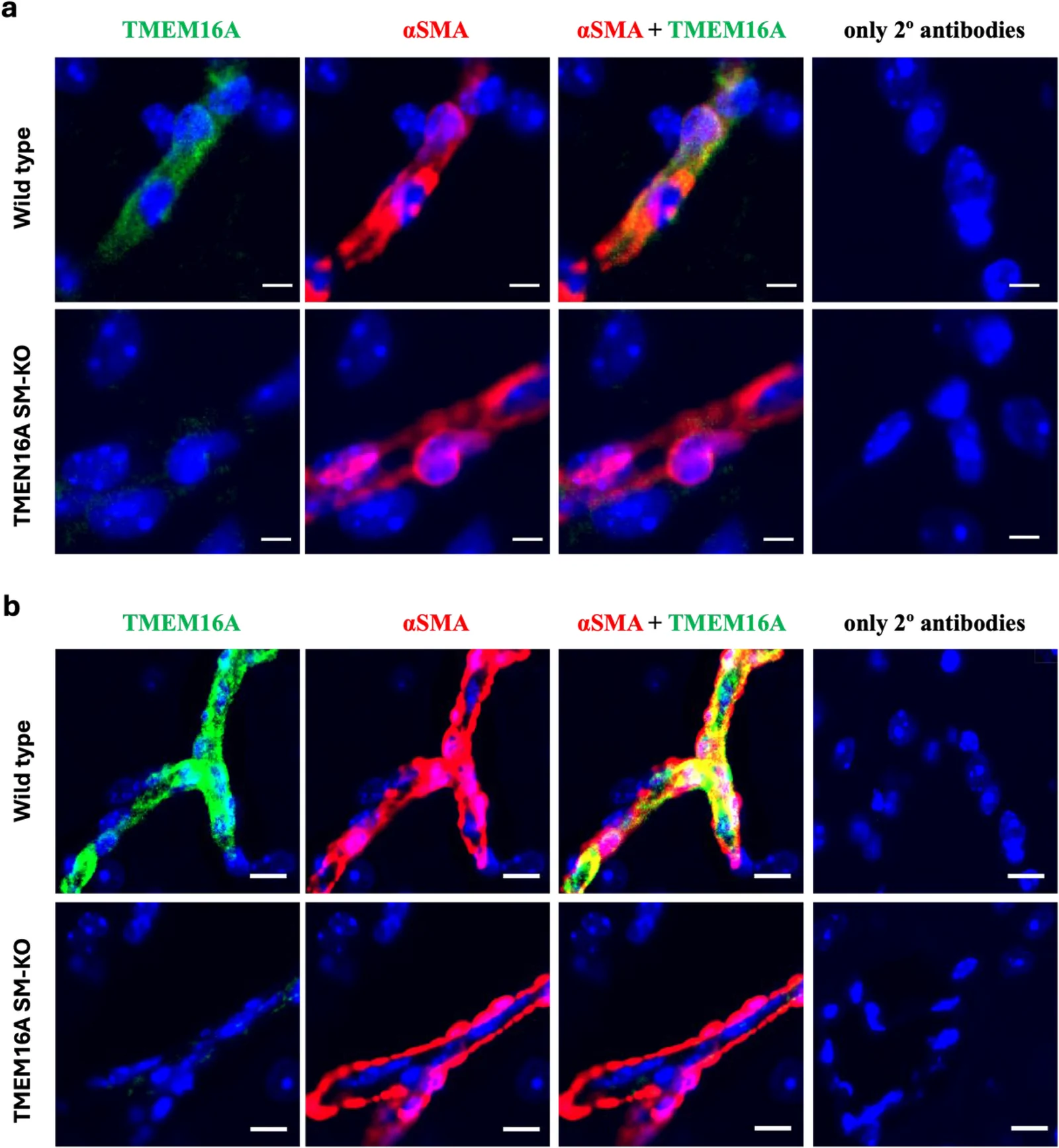
TMEM16A SM-KO mice lack TMEM16A in cerebrovascular mural cells. TMEM16A expresses in α-smooth-muscle-actin-positive pericytes (**a**) and arteriolar smooth muscle cells (**b**) in the brain parenchyma of WT mice but is absent in TMEM16A SM-KO mice. Brain slices were stained with TMEM16A targeting antibody (green), antibody against α-smooth-muscle-actin (red), as indicated, and DAPI (blue) to visualize cell nuclei. Staining is representative of 6 slices per group of 2 mice each; the negative control (only 2º antibody) is representative of 3 slices from WT and TMEM16A SM-KO mice each. All images are taken with the same settings. In (**a**), bars correspond to 5 μm, and in (**b**), bars correspond to 10 μm

Immunohistochemical staining of brain slices further demonstrates the expression of TMEM16A protein in α-smooth-muscle-actin positive pericytes (Fig. 2a) and arteriolar smooth muscle cells (Fig. 2b). TMEM16A SM-KO mice lack TMEM16A staining in their α-smooth-muscle-actin positive pericytes and arteriolar smooth muscle cells (Fig. 2).

**Fig. 3:**
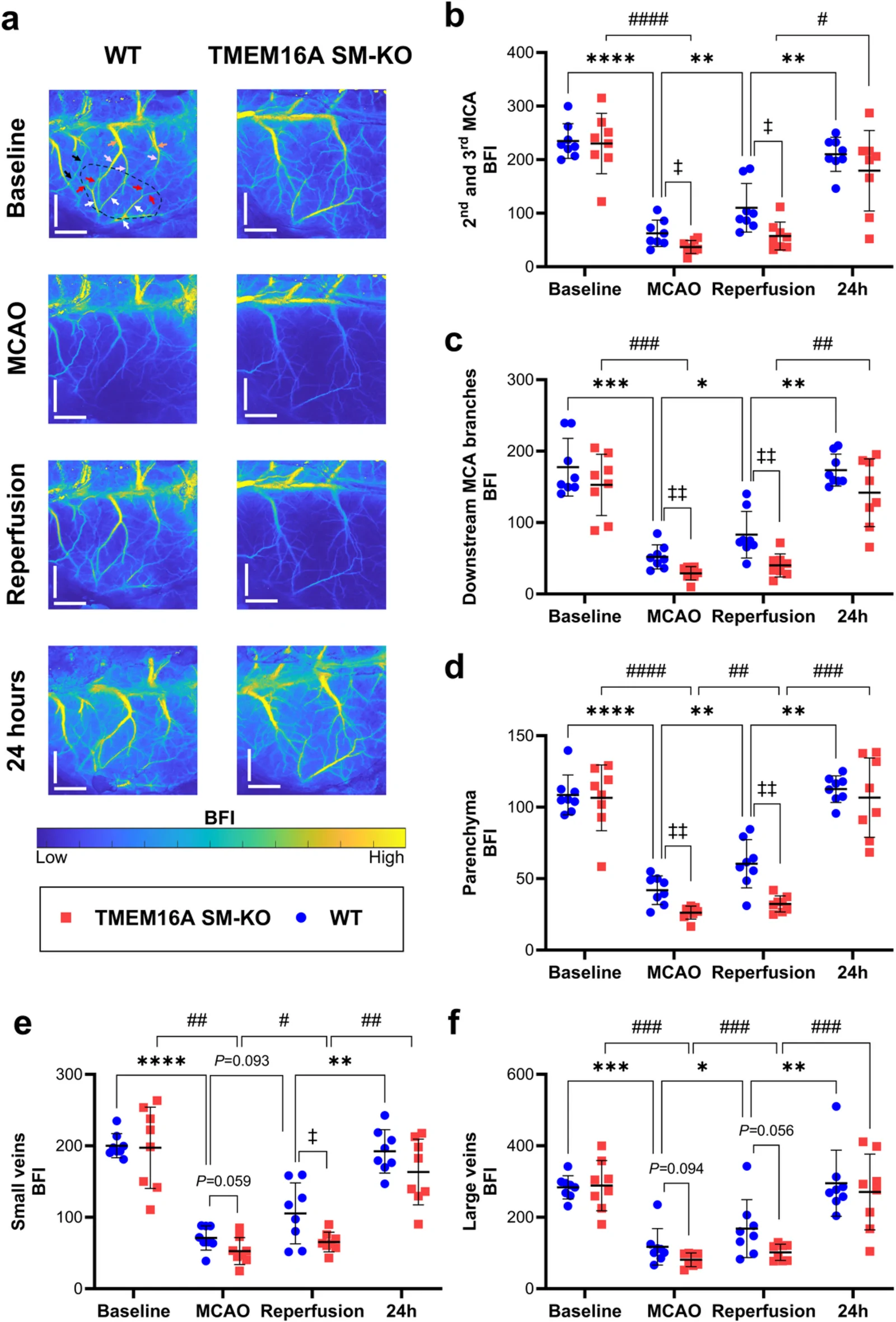
Transient middle cerebral artery occlusion (MCAO) is associated with a greater reduction in cerebral blood flow in TMEM16A SM-KO mice than in WT mice. Cerebral blood flow in the brain region supplied by the middle cerebral artery (MCA) was assessed with Laser Speckle Contrast Imaging at baseline, during MCAO, at reperfusion, and after 24 h of reperfusion in WT and TMEM16A SM-KO mice, as shown in representative images in (**a**). White scale bars indicate 1000 μm. BFI was measured in the 2^nd^ - and 3^rd^ -order branches of the MCA immediately downstream from the occlusion site (white arrows). Red arrows show the smaller MCA branches used for BFI measurements. Orange and pink arrows denote large and small draining veins, respectively. Black arrows indicate a branch of the anterior cerebral artery, and the dashed outline indicates the MCA-supplied parenchyma region that was analyzed. Averaged results for weighted BFI changes in 2^nd^ - and 3^rd^ -order MCA (**b**), smaller MCA branches (**c**), parenchyma (**d**), small veins (**e**), and large veins (**f**). *, **, ***, **** indicate *P* < 0.05, 0.01, 0.001, 0.0001 for intervention effect within the WT group. #, ##, ###, #### for *P* < 0.05, 0.01, 0.001, 0.0001 for intervention effect within the TMEM16A SM-KO group. ‡ and ‡‡ indicate *P* < 0.05 and < 0.01 for comparisons between WT and TMEM16A SM-KO at the same timepoint. Data were analyzed using two-way ANOVA followed by Tukey’s multiple comparisons test. *n* = 8 mice per group

Using Laser Speckle Contrast Imaging, cerebral perfusion was assessed in TMEM16A SM-KO and WT mice prior to and during MCAO, as well as during reperfusion for 24 h (Fig. 3a). Baseline BFI in different branches of middle cerebral arteries and in draining veins did not differ between genotypes. However, occlusion of the MCA resulted in a pronounced BFI reduction in TMEM16A SM-KO mice compared with WT. This effect was observed in both large (Fig. 3b) and small (Fig. 3c) downstream branches of the occluded MCA, as well as in parenchymal perfusion (Fig. 3d) and in both small (Fig. 3e) and large (Fig. 3f) draining veins. Immediately following occlusion release, reperfusion was attenuated in TMEM16A SM-KO mice in comparison with WT (Fig. 3b-f). Twenty-four hours after reperfusion, cerebral perfusion had largely recovered in both groups (Fig. 3b). When the diameters of the corresponding vessels were assessed at the same time points in the protocol, 2^nd^ and 3^rd^ order MCA diameters decreased during occlusion and immediately after reperfusion, possibly reflecting reduced transmural pressure downstream of the occlusion (Suppl. Figure 1a, b). In contrast, venous diameters did not change over this time (Suppl. Figure 1a, b). Importantly, no differences in vessel diameter were observed between genotypes.

Perfusion of the anterior cerebral artery, which is not directly exposed to the MCAO procedure, was also assessed by Laser Speckle Contrast Imaging (Suppl. Figure 2). BFI in the anterior cerebral artery was also significantly reduced during MCA occlusion and at the onset of reperfusion (Suppl. Figure 2a), whereas lumen diameter was unaffected by the intervention (Suppl. Figure 2b). Similar with the MCA, BFI reduction in the anterior cerebral artery was larger in TMEM16A SM-KO than in WT mice (Suppl. Figure 2a).

**Fig. 4:**
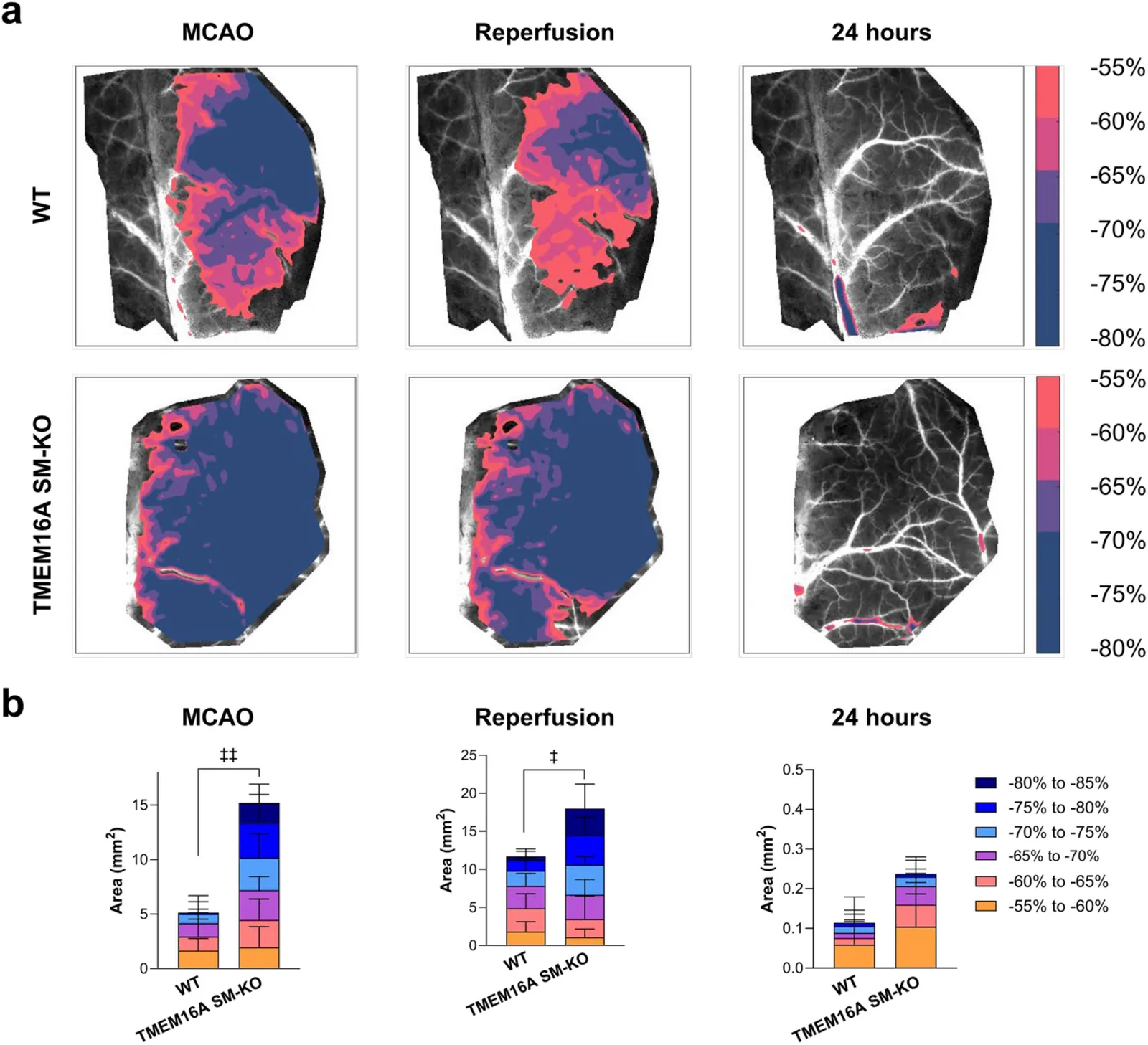
The degree of perfusion reduction is greater in TMEM16A SM-KO mice than in WT mice. (**a**) Percentage changes in BFI relative to baseline were assessed and quantified in 5% intervals to determine the spatial extent of perfusion reduction within the ipsilateral hemisphere of WT and TMEM16A SM-KO mice, as illustrated in representative images. (**b**) Averaged cortical areas exhibiting BFI reductions ranging from 55% to 80% quantified in 5% intervals during middle cerebral artery occlusion (MCAO), at the onset of reperfusion, and 24 h of reperfusion, as indicated. *n =* 6 + 6. ‡ and ‡‡ indicate *P* < 0.05 and < 0.01 as determined by two-way ANOVA followed by Sidak correction for multiple comparisons

The extent of the perfusion deficit across the ipsilateral hemisphere was assessed during MCAO, at the onset of reperfusion, and 24 h of reperfusion (Fig. 4a). Regions in which blood flow was reduced more than 75% during occlusion and more than 80% at onset of reperfusion were larger in TMEM16A SM-KO mice than in WT mice (Fig. 4b). In contrast, no difference was observed at 24 h of reperfusion (Fig. 4b).

**Fig. 5:**
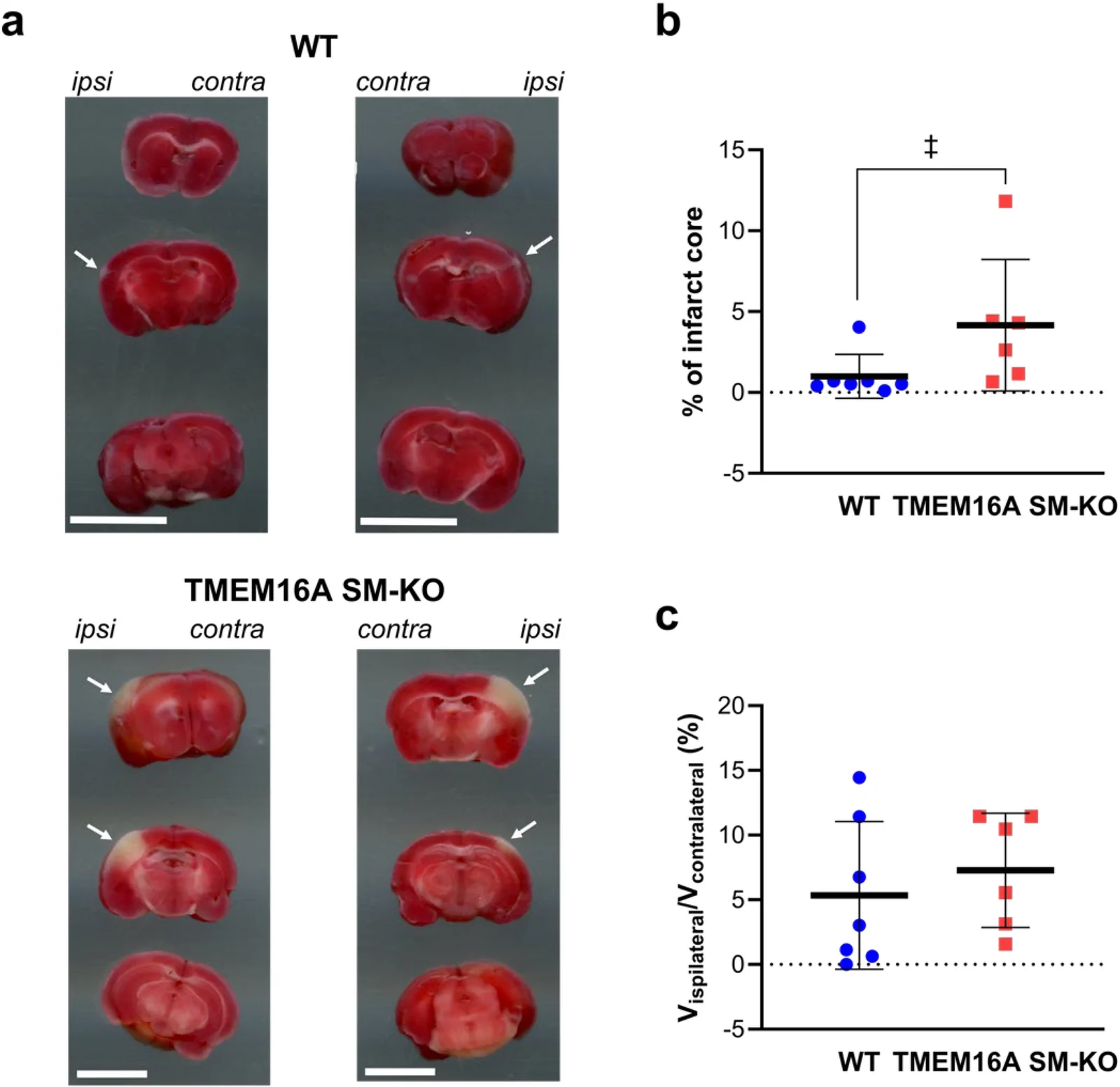
Infarct core volume was increased in TMEM16A SM-KO mice in comparison with WT mice 24 h after reperfusion. (**a**) Coronal brain slices. (2-mm thick) were stained with 2,3,5-triphenyltetrazolium chloride (TTC) and imaged from both sites. Three sliced sections encompassing most of the cerebrum were used for the analysis. Bars correspond to 5 mm. (**b**) Infarct core volume was calculated as a percentage of the total ipsilateral hemisphere volume. (**c**) Changes in the ipsilateral volume were assessed and expressed as a percentage of the contralateral hemisphere volume. *n* = 6–7. ‡ indicates *P* < 0.05, as determined by Mann-Whitney U test

The more pronounced perfusion deficit observed in TMEM16A KO mice (Figs. 2, 3 and 4) was reflected in the severity of stroke-reperfusion outcome, measured by infarct core volume 24 h after reperfusion (Fig. 5a). Infarct volume was larger in TMEM16A SM-KO mice compared with WT mice (Fig. 5b). Both genotypes demonstrated edema in the ipsilateral hemisphere; however, no difference in edema volume was detected between the genotypes (Fig. 5c).

**Fig. 6:**
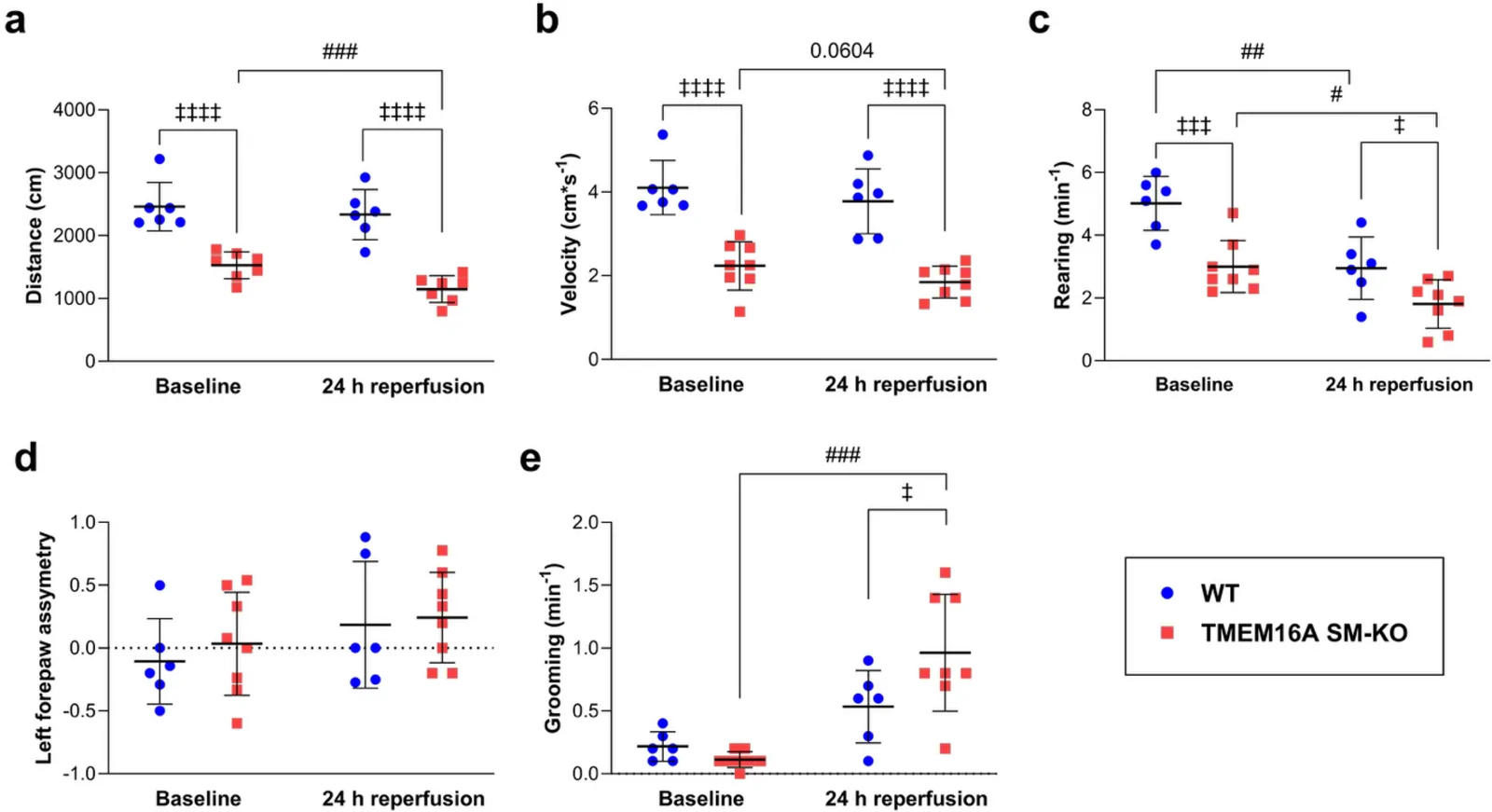
Locomotor function is more severely affected by ischemia-reperfusion in TMEM16A SM-KO mice than in WT mice. (**a**) Moving distance during a 10-min cylinder test at baseline and 24 h after reperfusion. (**b**) Average movement velocity during the test. (**c**) Rearing events per minute. (**d**) Left forepaw asymmetry. (**e**) Grooming event per minute. *n =* 6 + 6. ** indicate *P*< 0.01 within the WT mice. #, ## and ### indicate *P* < 0.05, < 0.01 and < 0.001 within the TMEM16S KO mice. ‡, ‡‡‡ and ‡‡‡‡ indicate *P* < 0.05, < 0.001 and < 0.0001 for comparison between genotypes. Data were analyzed using two-way ANOVA followed by Sidak’s multiple comparisons test

To evaluate the functional consequences of ischemia-reperfusion in WT and TMEM16A SM-KO mice, locomotor activity was assessed before MCAO and 24 h after reperfusion (Fig. 6). Surprisingly, TMEM16A SM-KO mice had reduced locomotor activity at baseline, as indicated by reduced total moving distance (Fig. 6a), lower movement velocity (Fig. 6b), and fewer rearing events (Fig. 6c). These genotype-dependent differences persisted after ischemia–reperfusion (Fig. 6a-c). Following ischemia-reperfusion, WT mice did not change their distance and velocity of movement, whereas TMEM16A SM-KO mice demonstrated a reduction in distance traveled (Fig. 6a). Rearing activity was reduced after ischemia–reperfusion in both genotypes, yet the difference between WT and TMEM16A SM-KO remained (Fig. 6c). No effect of the genotype or ischemia–reperfusion was detected in the measured forepaw asymmetry (Fig. 6d). Interestingly, grooming behavior increased 24 h after reperfusion in both groups, with a pronounced increase in TMEM16A SM-KO in comparison with WT mice under post-ischemic conditions (Fig. 6e). Correlation analyses between arterial BFI and locomotor parameters (Suppl. Figure 3) revealed heterogeneous associations across behavioral measures, genotypes, and time points. In WT mice, baseline arterial BFI showed significant positive correlations with distance traveled and velocity, whereas these associations were weaker or absent at 24 h post-stroke and in TMEM16A SM-KO mice at any time. Other behavioral parameters, including rearing, grooming, and left forepaw asymmetry, did not demonstrate consistent relationships with arterial BFI. Overall, these findings suggest that variability in cerebral perfusion may partially contribute to locomotor performance but do not fully account for the observed behavioral differences between experimental groups.

**Fig. 7:**
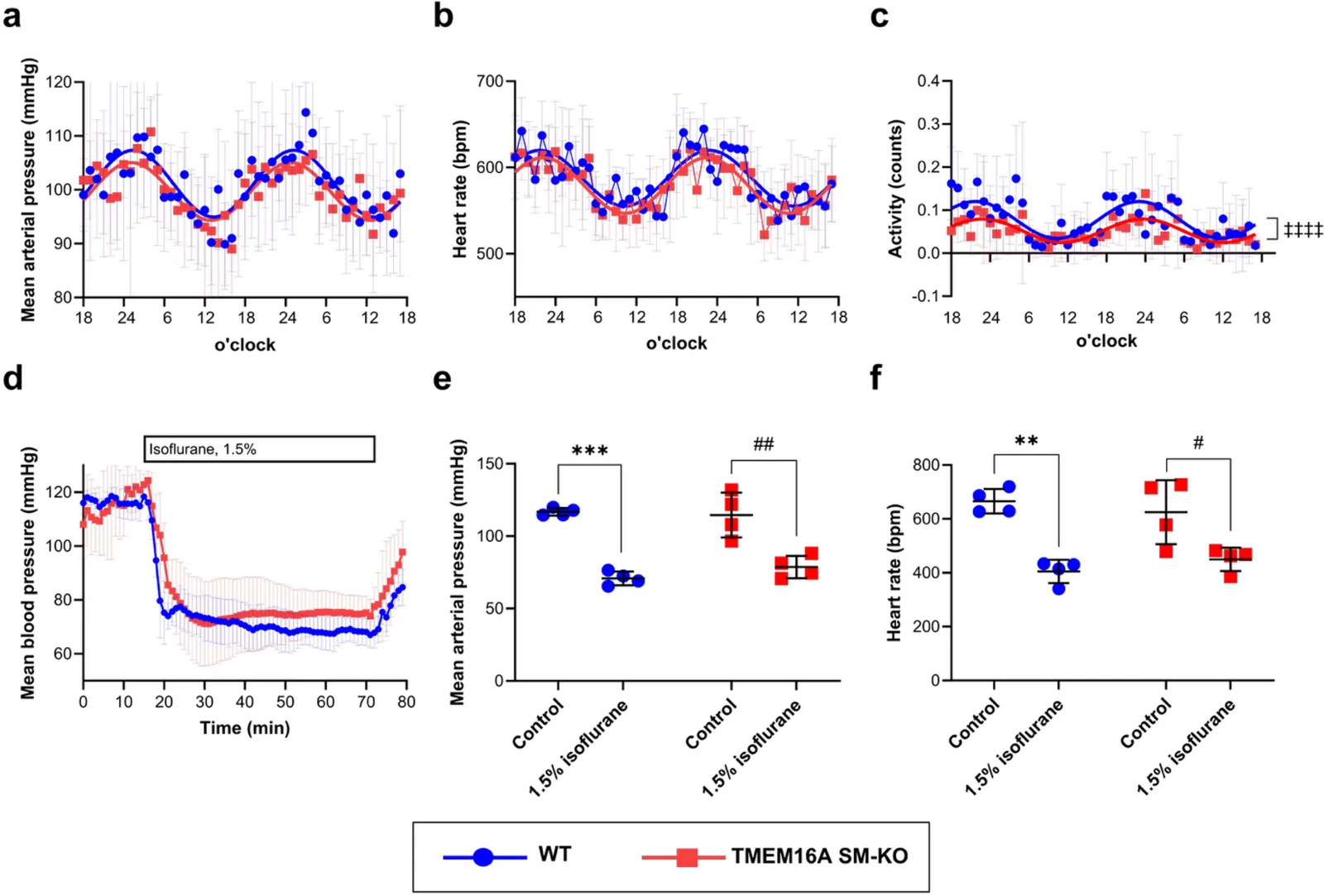
Basic cardiovascular parameters do not differ between TMEM16A SM-KO and WT mice. Mean arterial pressure (**a**) and heart rate (**b**) were measured radiotelemetrically over 48 h, demonstrating normal circadian variation, with no difference between WT (*n* = 5) and TMEM16A SM-KO (*n* = 8) mice. See also Suppl. Figure 4. Locomotor activity (**c**) recorded radiotelemetrically demonstrated reduced activity (*P* < 0.0001, extra sum-of-squares *F* test) of TMEM16A SM-KO mice (*n* = 9) compared with WT mice (*n* = 6). Isoflurane anesthesia (1.5%) produced a stable reduction in blood pressure (**d** and **e**) and heart rate (**f**) in both TMEM16A SM-KO (*n* = 4) and WT mice (*n* = 4), yet no genotype-dependent difference was observed. See also Suppl. Figure 6. ‡, ‡‡, and ‡‡‡ indicate *P* < 0.05, < 0.01, and < 0.001 by two-way ANOVA followed by Fisher’s LSD test

A potential explanation for the observed differences between genotypes could be due to a variation in blood pressure. However, the radiotelemetry measurements showed no difference in blood pressure between WT and TMEM16A SM-KO mice (Fig. 7a, b, and Suppl. Figure 4). Consistent with other behavioral measurements, TMEM16A SM-KO mice were less locomotory active than WT mice (Fig. 7c). As stroke intervention and brain perfusion imaging were performed under isoflurane anesthesia, and differential sensitivity to isoflurane can contribute to the experimental outcomes [41], the cardiovascular effect of isoflurane was evaluated. Light isoflurane anesthesia (0.6%) produced only a modest suppression of blood pressure and heart rate, with no difference between genotypes (Suppl. Figure 5). Increasing isoflurane concentration to 1.5% resulted in a pronounced suppression of cardiovascular parameters; however, no genotype-dependent difference was observed (Fig. 7d-f and Suppl. Figure 6).

**Fig. 8:**
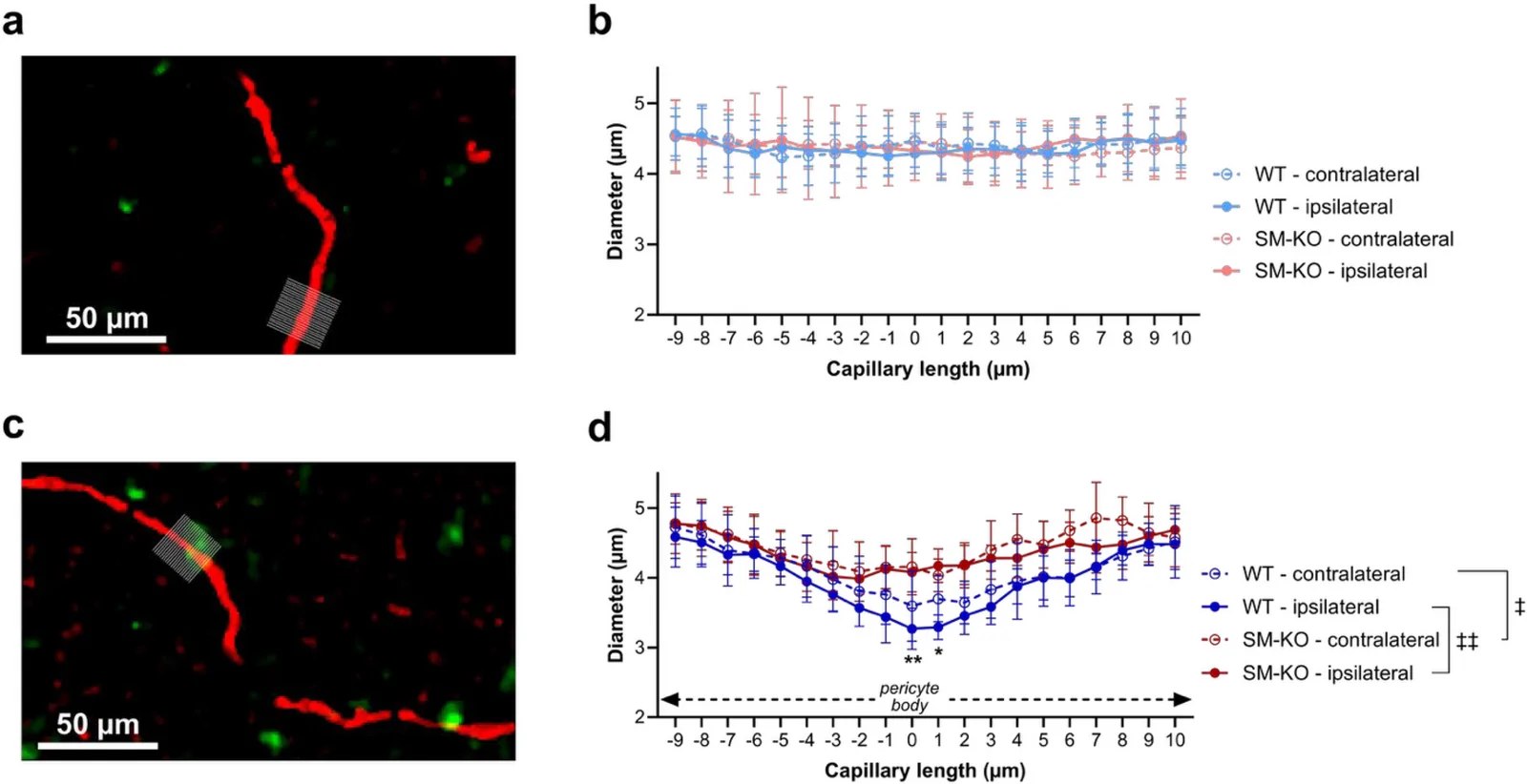
TMEM16A SM-KO pericytes exhibit reduced contractile tone and a lack of its ischemia-induced potentiation. Capillaries in the brain slices obtained 24 h after reperfusion were labeled with lectin (red), and pericytes were labeled with PDGFRβ antibody (green). (**A**) Representative image of pericyte-free capillary assessed using the VasoMetrics tool. (**B**) Averaged diameters of pericyte-free capillaries in the ipsilateral and contralateral hemispheres from WT and TMEM16A SM-KO mice as indicated. (**C**) Representative image of a capillary with an associated pericyte assessed using the VasoMetrics tool. (**D**) Averaged diameters of pericyte-associated capillaries in the ipsilateral and contralateral hemispheres from WT and TMEM16A SM-KO mice. *n =* 6–7 per group. ‡ and ‡‡, *P* < 0.05 and < 0.01 between the genotypes, compared with two-way ANOVA followed by Sidak correction for multiple comparison test

The question of whether stroke severity correlated with brain vascular density was examined. Capillary bed morphology did not differ between genotypes in matching cortex regions supplied by the middle cerebral artery (Suppl. Figure 7a-c). Although both genotypes exhibited less microvascular density in the ipsilateral cortex compared with the contralateral hemisphere 24 h after stroke-reperfusion, no genotype-dependent difference was detected. Although post-mortal measurements could be affected by changes in cell volume, similar capillary capacity reduction in peri-infarct cortex 24 h after stroke was previously reported in vivo [42]. Microvascular lacunarity was also similar across cortex regions examined (Suppl. Figure 7d). Assessment of capillary diameter revealed no difference between genotypes or hemispheres for capillary segments without associated pericytes (Fig. 8a, b). In contrast, capillary diameter near the adjacent pericyte soma was reduced in both WT (*P* < 0.0001) and TMEM16A SM-KO mice (*P* = 0.017; Fig. 8c, d). Notably, the diameter of pericyte-associated capillaries in the ipsilateral hemisphere was profoundly smaller in WT mice than in TMEM16A SM-KO mice (*P* = 0.0002; Fig. 8d). Similar smaller diameters of pericyte-associated capillaries were observed in the contralateral hemisphere of WT mice compared with matching capillaries of TMEM16A SM-KO mice (*P* = 0.0219; Fig. 8d).

**Fig. 9:**
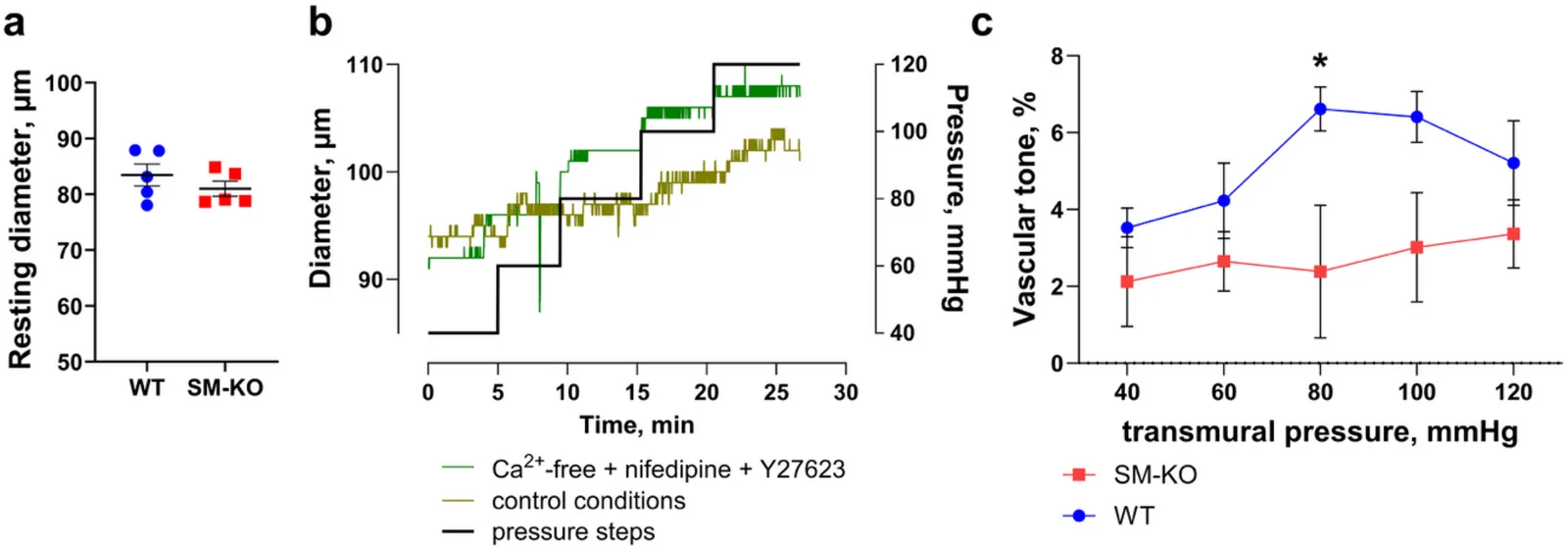
Middle cerebral arteries from TMEM16A SM-KO mice showed diminished vascular tone ex vivo. Pressurized at 60 mmHg, the arteries from WT and TMEM16A SM-KO mice had similar outer diameter (**a**). Data were compared with Welch’s *t*-test. Arteries were exposed for 5 min to different transmural pressures between 40 and 120 mmHg with 20 mmHg increments, and their diameters were assessed under control conditions and after they were relaxed in Ca^2+^-free PSS with 10 µM nifedipine and 10 µM Y27623; (**b**) for representative traces and (**c**) for averaged data. Data were compared with two-way ANOVA followed by Sidak correction for multiple comparisons. *, *P* < 0.05. *n* = 5

To elucidate whether TMEM16A omission in mural cells affects their ability to autoregulate cerebrovascular diameter, we analyze myogenic tone in pressurized middle cerebral arteries isolated from WT and TMEM16A SM-KO mice (Fig. 9). Pressurized at 60 mmHg arteries from WT and TMEM16A SM-KO mice had similar outer diameters (Fig. 9a). Arteries were then exposed to intraluminal pressures between 40 and 120 mmHg with 20 mmHg increment under control conditions and after they were fully relaxed (Fig. 9b). Vascular tone was larger in WT middle cerebral arteries than in TMEM16A SM-KO group with significant difference (*P* = 0.032) at 80 mmHg (Fig. 9c).

**Fig. 10:**
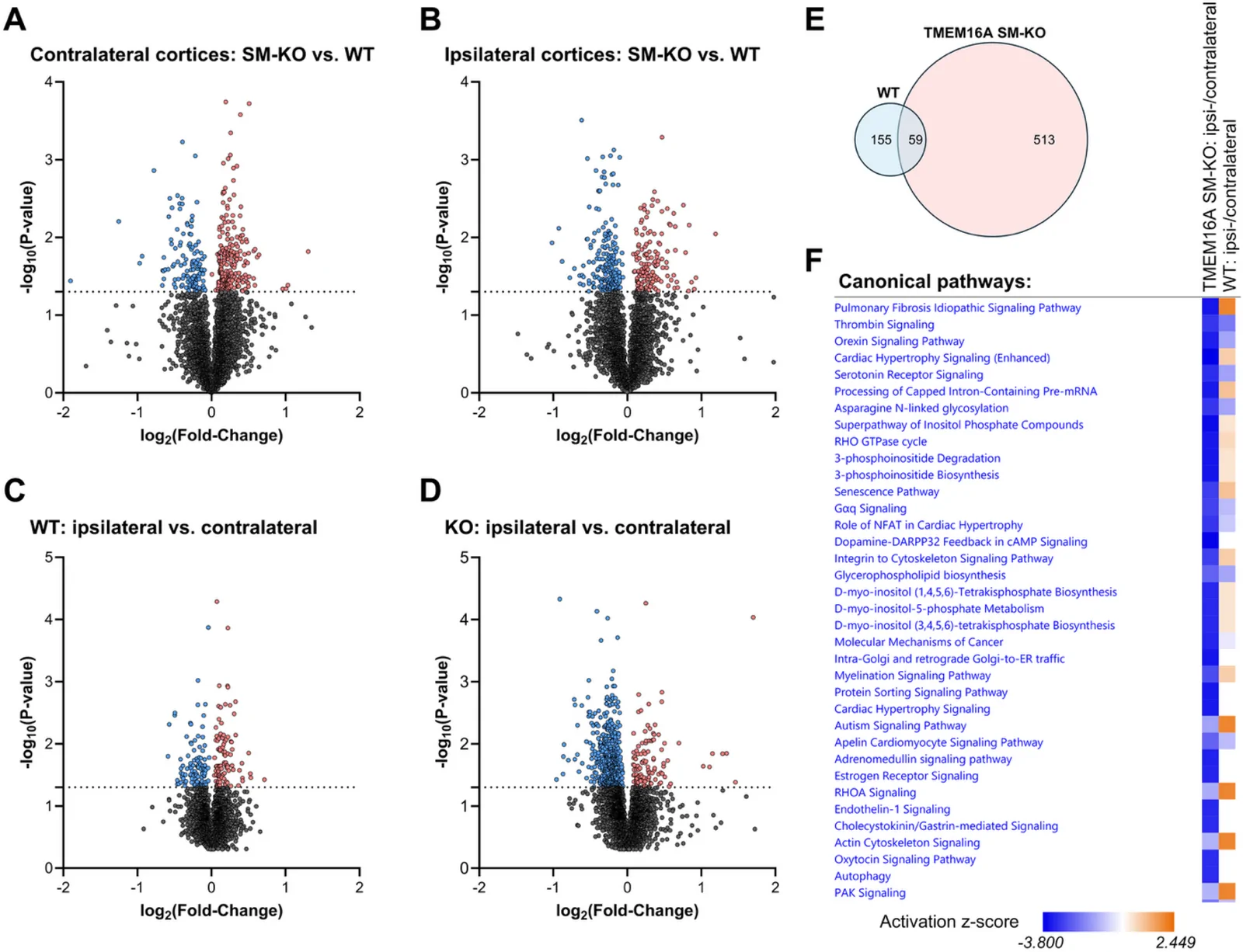
Proteomics analysis reveals greater ischemia-reperfusion-induced expression changes in TMEM16A SM-KO cortices than in WT cortices. Proteomics profiling was performed matching contralateral (**a**) and ipsilateral cortices (**b**) from TMEM16A SM-KO (*n =* 5) and WT (*n =* 4) mice, or within the specific genotype comparisons of ipsilateral and contralateral cortices in WT (**c**) or TMEM16A SM-KO (**d**) mice. Significantly downregulated proteins are indicated by blue, whereas red indicates upregulated protein expression. See also Suppl. Table 2. (**e**) Venn diagram of overlapping differentially expressed proteins in ipsilateral vs. contralateral cortices detected in WT and TMEM16A SM-KO mice. (**f**) Canonical pathway alterations between the ipsilateral and contralateral cortices in TMEM16A SM-KO and WT mice, demonstrating a pronounced effect in TMEM16A SM-KO mice. See also Suppl. Tables 3 and Suppl. Figure 8 for the complete dataset. *P* < 0.05 was considered significant for either unpaired (**a**, **b**) or paired (**c**, **d**) *t*-test, as appropriate

Proteomics analysis of ipsilateral and contralateral cortices from WT and TMEM16A SM-KO mice identified a total of 5,160 proteins. Of these, 350 proteins were differentially expressed when contralateral cortices from the two genotypes were compared, and 343 proteins were differentially expressed when the corresponding ipsilateral cortices were compared (Suppl. Tables 1 and 2, Fig. 10a, b). When paired ipsilateral and contralateral cortices were compared within the same genotype, 214 and 572 differentially expressed proteins were detected in WT and TMEM16A SM-KO mice, respectively (Suppl. Tables 1 and 2, Fig. 10c, d).

Ingenuity Pathway Analysis comparing contralateral cortices from TMEM16A SM-KO and WT mice predicted an upregulation of inflammatory pathways, along with alterations in synaptic transmission and ion transport in TMEM16A SM-KO mice (Suppl. Table 3, Suppl. Figure 8, and Fig. 10f). The analysis further suggested congenital brain abnormalities, with predicted potentiation of synaptogenesis signaling and long-term potentiation pathways in TMEM16A SM-KO mice.

Comparisons of ipsilateral cortices revealed potentiation of inflammatory, apoptotic, and stress-response pathways in TMEM16A SM-KO mice relative to WT mice, whereas pathways related to synaptic function and smooth muscle contraction, including α-adrenergic signaling, were predicted to be suppressed (Suppl. Table 3, Suppl. Figure 8, and Fig. 10f). Increased expression of NFE2L2 (also known as NRF2) suggests activation of mechanisms associated with neurodegeneration processes and cellular defense responses. As these results are based on bulk proteomics, caution should be exercised in their interpretation with respect to mural cell function, as the contribution of mural cell proteins is relatively small because of a bulk signal dilution. Nevertheless, suppression of ARHGEF7 may suggest decreased activation of Ras-like Rho GTPases, potentially linked to reduced myosin light chain phosphorylation and suppressed contractile activity of mural cells.

Within the WT genotype, comparisons of ipsilateral and contralateral cortices revealed relatively modest pathway changes, primarily involving activation of RAF/MAP kinase and glutamatergic signaling, as well as Hypoxia-Inducible Factor 1α signaling (Suppl. Table 3, Suppl. Figure 8, and Fig. 10f). Ischemia-reperfusion was also associated with suppression of calcium signaling; however, this effect was observed in both genotypes.

In contrast, TMEM16A SM-KO mice demonstrated pronounced suppression of multiple pathways in the ipsilateral cortex in comparison with the contralateral cortex, including α-adrenergic, serotonergic, endothelin, insulin receptor tyrosine kinase, and glutaminergic signaling, as well as mitochondrial function and several additional pathways (Suppl. Table 3, Suppl. Figure 8, and Fig. 10f). These changes were associated with predicted involvement of cytokines IL4 and IL5 and their upstream modulators, including TFAM and CD38. Reduced activity of YAP1 and SRSF1, regulators of cell motility and invasiveness, suggested alterations in lipid metabolism and links between cellular motility and metabolic state. Overall, ischemia-reperfusion in TMEM16A SM-KO mice was associated with broad suppression of long-term synaptic depression and inflammatory signaling.

## Discussion

This study examined whether TMEM16A-associated Ca²⁺-activated Cl⁻ conductance in cerebral mural cells plays a detrimental role in stroke outcome, as previously proposed based on studies using putative TMEM16A inhibitors to limit ischemia-induced perfusion decline and stroke severity [13, 28]. To address this question, an inducible mouse model lacking TMEM16A selectively in smooth muscle myosin heavy chain (Myh11)-expressing cells was generated, thereby targeting both vascular smooth muscle cells [43] and capillary pericytes [44], collectively called mural cells. Loss of TMEM16A protein was accompanied by diminished Ca²⁺-activated Cl⁻ current in cerebrovascular smooth muscle cells, confirming effective gene deletion.

Previous studies have identified TMEM16A as an important amplifier of cerebrovascular contraction, coupling depolarizing Ca²⁺-dependent Cl⁻ efflux to voltage-gated Ca²⁺ influx [17, 18]. This model has been supported by in vivo and ex vivo studies employing pharmacological modulation [2, 5–12, 14–16, 45, 46] as well as genetic manipulation of TMEM16A in the vascular wall [3, 4, 13, 47, 48], demonstrating the importance of TMEM16A for myogenic and agonist-induced contraction, whereas K⁺-induced depolarization was largely unaffected. However, this relationship has not been entirely consistent. Several models of TMEM16A downregulation paradoxically exhibited enhanced vascular contraction, likely due to compensatory alterations in Ca²⁺-signaling proteins [33, 48]. These observations indicate that both degree and mode of TMEM16A manipulation critically influence smooth muscle cell phenotype and function. This distinction is particularly relevant because several TMEM16A knockout models display a loss of Ca²⁺-activated Cl⁻ conductance without complete protein elimination [4]. In the present study, TMEM16A was deleted by excision of exon 5, in contrast to exon 21 deletions previously reported [4], resulting in complete loss of both TMEM16A protein and Ca²⁺-dependent Cl⁻ currents in smooth muscle cells.

Given its unique ability to amplify Ca²⁺ signaling and vascular tone, TMEM16A has emerged as a key regulator of both arteriolar smooth muscle cells and capillary pericytes [4, 15, 16, 49]. Despite TMEM16A deletion in SMMHC–expressing cells previously being reported to reduce systemic blood pressure [4], no such effect was observed in the present study or in prior work examining TMEM16A downregulation [33]. This discrepancy likely reflects the different functional significance of TMEM16A across vascular beds [4, 50], where both suppression [4, 47, 48] and potentiation [33, 48] of vascular contractility have been reported, as well as in blood vessels of different caliber [13].

Recent expression analyses suggest that within the brain microvasculature, TMEM16A is more abundantly expressed in capillary pericytes than in arteriolar smooth muscle cells, where its contribution to agonist-induced contraction appears modest [4, 13]. In the present study, we did not quantify TMEM16A expression levels in cerebrovascular smooth muscle cells and pericytes, although its presence has been demonstrated in both vascular cell types. Control of cerebrovascular resistance has largely been attributed to ensheathing pericytes [13]. However, a systematic evaluation of TMEM16A expression and its functional relevance across distinct pericyte subtypes remains lacking [19, 51]. In the present study, no distinction was made between capillary pericyte subtypes. Both cerebrovascular smooth muscle cells [52] and pericytes [53–55] contribute to the generation of myogenic tone and the regulation of cerebral perfusion. In addition to the well-established myogenic responses in small arteries [52], pericytes ensheathing the first three to four generations of capillary branches have been shown to constrict in response to increased pressure [53–55]. Consistent with this, our postmortem immunohistochemical analyses suggest relaxation of capillary pericytes in TMEM16A SM-KO mice, whereas WT mice maintained basal pericytic tone, supporting a role for TMEM16A in cerebral autoregulation. Although this approach has inherent limitations, particularly the absence of transmural pressure and postponed fixation, this interpretation is further supported by the observed reduction of myogenic tone in middle cerebral arteries from TMEM16A SM-KO mice, indicating an overall impairment of cerebral myogenic autoregulation in these animals. Furthermore, a previous study comparing in vivo capillary flow with postmortem capillary diameter measurements suggested that 24 h after ischemia–reperfusion, postmortem narrowing of capillaries ensheathed by pericytes is associated with reduced capillary perfusion, increased capillary staling, and prolonged transit time, despite normalization of blood flow in the large surface arteries [42].

Unexpectedly, TMEM16A deletion was associated with a worsened stroke outcome, manifested as impaired reperfusion dynamics, enlarged infarct core, and reduced motor performance compared with matched WT controls. These effects could not be attributed to differences in baseline cardiovascular parameters or altered sensitivity to isoflurane anesthesia. Instead, we propose that the absence of TMEM16A impairs cerebrovascular autoregulation, reducing the efficiency of cerebral blood flow redistribution and thereby exacerbating ischemic injury. Consistent with this interpretation, perfusion in the anterior cerebral artery, which supplies regions distinct to the occlusion site, was also reduced. Accordingly, we report diminished myogenic tone in TMEM16 SM-KO middle cerebral arteries compared with controls. This impaired ability to redistribute blood flow to brain regions according to local metabolic demands may underlie both the reduced baseline activity, reflecting compromised neurovascular coupling, and the pronounced reduction in cerebral perfusion during stroke due to limited capacity to shunt blood toward hypoperfused regions.

It has also been proposed that ischemia induces endothelin-1 release, activating TMEM16A and producing sustained pericyte contraction that restricts post-ischemic blood flow [13]. In line with this model, topical pharmacological blockade of TMEM16A was previously reported to reduce capillary staling, enhance cerebral blood flow, and limit infarct size [13]. In the present study, the deletion of TMEM16A from mural Myh11-expressing cells resulted in worse stroke outcomes. This discrepancy likely reflects differences in experimental stroke models and the way of interference with TMEM16A. Prior studies have employed severe experimental interventions to model global cerebral hypoperfusion, including bilateral common carotid artery occlusion in vivo and oxygen–glucose deprivation in brain slice preparations [13]. In contrast, the current study employed focal occlusion of a distal MCA branch, the model that preserves a greater capacity for autoregulatory blood flow redistribution during reperfusion. These compensatory processes appear to depend critically on intact pericyte contractility and overall cerebrovascular regulatory function [56]. Importantly, this study supports the previous conclusion [13] that TMEM16A plays an important role in mural cell regulation of local perfusion and that enhanced TMEM16A signaling can worsen stroke outcome. However, our findings further suggest that such effects should be targeted locally in order to preserve cerebral autoregulation. Together, these previous [13] and current findings suggest that local rather than systemic use of TMEM16A inhibitors may represent a promising therapeutic strategy in stroke, for example, through catheter-based drug delivery during thrombectomy.

Autoregulatory mechanisms of small arteries and capillary pericytes contribute to the redistribution of cerebral blood flow [21–23, 57]. Constriction in one region can redirect the blood flow, whereas failure of this autoregulatory mechanism may produce widespread perfusion deficits despite local vasodilation [58]. Such ‘blood-stealing’ phenomena may be particularly deleterious in ischemic tissue. The current study proposes that this mechanism underlies the worsened stroke outcome observed in TMEM16A knockout mice. The loss of TMEM16A in pericytes and smooth muscle cells abolishes cerebrovascular autoregulation, resulting in insufficient reperfusion of ischemic regions and diversion of blood flow to non-ischemic areas, including the contralateral hemisphere. Dynamic regulation of cerebrovascular resistance is also thought to stabilize cellular metabolism [59]. Blood stealing may reduce local perfusion pressure, promote capillary staling, and impair microvascular network connectivity and anastomotic formation [60]. Accordingly, loss of autoregulation, whether due to aging [60] or TMEM16A deletion as in the current study, may increase capillary flow heterogeneity and compromise tissue oxygenation, maybe already at baseline, yet particularly during reperfusion [61, 62]. Consistent with this interpretation, proteomic analysis revealed enhanced ischemia-induced suppression of synaptogenesis and of the canonical GABAergic, glutamatergic, and endocannabinoid pathways.

Although the bulk proteomics data should be interpreted with caution due to substantial dilution of the mural cell signal, the findings may suggest a potential phenotypic shift in mural cells of TMEM16A SM-KO mice, consistent with previous reports implicating TMEM16A in transcriptional regulation [63, 64]. TMEM16A has also been implicated in stroke-related vascular remodeling [28, 65] and proposed to negatively regulate cerebrovascular hypertrophy by arresting the cell cycle [29]. A deletion of TMEM16A has been shown to suppress the STAT3 transcription factor, important for downstream MAPK and RhoA pathways [32]. These pathways are modified differently in the ipsilateral hemisphere cortices of WT and TMEM16A SM-KO mice. In the contralateral hemispheres of TMEM16A SM-KO mice, activation of the RAF/MAPK signaling was observed. Among its other important functions, this signaling is associated with a change in mural cells from a contractile to a proliferative/migratory phenotype [66]. Concurrent suppression of canonical Rho family GTPase signaling, which is essential for mural cell contractility, was also evident in the proteomics dataset. TMEM16A has been shown to modulate RhoA/ROCK signaling [9], potentially via WNK1 kinase, thereby sensitizing the contractile machinery to calcium [67]. Interestingly, several pro-contractile pathways, including Gαi, Gα12/13, MAPK6/MAPK4, and p38 MAPK signaling, were predicted to be activated, possibly reflecting compensatory responses to loss of vascular tone in cerebral microcirculation. The compensatory nature of these changes is further supported by analysis of ipsilateral hemispheres, in which these pathways were suppressed in TMEM16A SM-KO mice. Reduced α-adrenergic signaling, diminished ERK/MAPK, PI3K/AKT, and RhoA activity, together with increased protein kinase A signaling, may point toward a net suppression of contractility underlying the observed loss of cerebrovascular tone.

In the present study, all BFI measurements were performed under 1.5% isoflurane in 100% oxygen. Isoflurane is a potent vasodilator, and the reported BFI measurements do not thereby reflect the awake or normoxic state. Despite all groups being assessed under identical conditions, this remains an important confounder for physiological and translational interpretation. The observed differences in several locomotor parameters between WT and TMEM16A SM-KO mice at baseline suggest that TMEM16A deletion may influence aspects of animal physiology or behavior independent of ischemic injury. Additional correlation analyses between arterial BFI and behavioral outcomes demonstrated heterogeneous and inconsistent associations across parameters and time points, indicating that cerebral perfusion changes alone do not fully explain the observed behavioral variability. Given the role of TMEM16A in regulating cerebrovascular tone and contractility [3, 13], subtle alterations in cerebrovascular responsiveness and neurovascular coupling could potentially influence locomotor performance even in the absence of ischemic injury. Future studies will be required to further characterize potential baseline phenotypes associated with TMEM16A deficiency. The mechanistic interpretations are based on bulk cortical proteomics, and contributions from non-mural cell populations cannot be excluded. TMEM16A inhibition has previously been reported to reduce infarct size by preserving blood–brain barrier integrity through endothelial mechanisms [28]. However, in the present model, TMEM16A deletion was restricted to mural cells, supporting their dominant role in determining stroke outcome. This mouse model does not permit the use of females or enable detailed differentiation between distinct cerebral mural cell types. Addressing these mechanistic aspects in greater depth will therefore be the focus of future studies. In accordance with STAIR (Stroke Therapy Academic Industry Roundtable) recommendations [68], future studies should validate these findings across additional experimental models, species, and both sexes, and carefully evaluate pharmacological modulation to distinguish mural cell–specific effects from those in other cell types.

In conclusion, loss of TMEM16A-mediated Ca²⁺-activated Cl⁻ conductance in mural cells worsens stroke outcome, likely due to impaired autoregulation in cerebral circulation. The resulting loss of cerebrovascular tone compromises cerebral autoregulation, reducing the efficiency of blood flow redistribution and capillary flow homogeneity during reperfusion. These findings identify TMEM16A as a critical determinant of stroke recovery and suggest that while localized suppression of TMEM16A activity may alleviate peri-infarct vasospasm, global or systemic inhibition may have adverse consequences.

## Supplementary Information

Below is the link to the electronic supplementary material.


Supplementary Material 1 (XLSX 3.54 MB)



Supplementary Material 2 (XLSX 1.06 MB)



Supplementary Material 3 (XLSX 64.4 KB)



Supplementary Material 4 (DOCX 3.92 MB)



Supplementary Material 5 (PDF 6.21 MB)


## Data Availability

All data, materials, and custom code supporting the findings of this publication are available from the corresponding author upon reasonable request.

## Declarations

### Data Transparency

All data, materials, and custom code supporting the findings of this publication are available from the corresponding author upon reasonable request.
