# Supplementary material for: Loss of Vascular TMEM16A Impairs Cerebral Autoregulation and Exacerbates Ischemia–reperfusion Injury
Source: Transl Stroke Res. 2026 Jul 22;17(4):85. doi: 10.1007/s12975-026-01471-4 (PMC13391751; doi:10.1007/s12975-026-01471-4)
Supplement: Supplementary file 4 — Supplementary Material 4 (DOCX 3.92 MB) [file 12975_2026_1471_MOESM4_ESM.docx]

Supplementary

Title

Loss of vascular TMEM16A impairs cerebral autoregulation and exacerbates ischemia–reperfusion injury.

Authors

Ask Carit Andersen^1^, Ida Damsgaard Larsen^1^, Elizaveta Melnikova^1^, Marlon Gernemann^1^, Boris V. Skryabin^2^, Tina Myhre Pedersen^1^, Hans Christian Beck^3^, Halvor Østerby Guldbrandsen^1,4^, Eugenio Gutierrez^5^, Christian Aalkjær^1^, Dmitry D. Postnov^5^, Vladimir V. Matchkov^1^, Line Mathilde Brostrup Hansen^1^

Affiliations

^1^ Department of Biomedicine, Health, Aarhus University, Aarhus, Denmark

^2^ Medical Faculty, Core Facility Transgenic Animal and Genetic Engineering Models (TRAM), University of Muenster, Muenster, Germany

^3^ Department for Clinical Biochemistry, University of Southern Denmark, Odense, Denmark

^4^ Department of Clinical Medicine, Aarhus University, Aarhus, Denmark

^5^ Center of Functionally Integrative Neuroscience, Department of Clinical Medicine, Aarhus University, Aarhus, Denmark

Overview

Supplementary materials and methods Page: 2-8

Supplementary figures Page: 9-16

Supplementary table legends Page 17

Supplemental materials and methods

Ethical statement

All animal experiments conformed to the guidelines from the European Communities Council Directive (86/609/EEC) for the Protection of Animals used for Experimental and other Scientific Purposes. Permission was granted from the Animal Experiments Inspectorate of the Danish Ministry of Environment and Food.

Experimental animals and generation of smooth muscle cell-specific TMEM16A knockout mice

Nineteen WT control and twenty-eight TMEM16A SM-KO male mice, aged 3-5 months, were utilized in this study. Mice with TMEM16A SM-KO specific for the cells expressing smooth muscle myosin heavy chain (Myh11), i.e., smooth muscle cells and pericytes, were generated by flanking exon 7 of the TMEM16A gene with loxP sites using a previously described construct *[1]*. Floxed TMEM16A mice were backcrossed on the C57BL/6JRj background for 10 generations and subsequently crossed for seven generations with SMMHC-Cre^ERT2^ mice, which express tamoxifen-inducible Cre recombinase under the transcriptional control of the Myh11 promoter *[2]*. The knockout mice carried the floxed TMEM16A allele and expressed fusion proteins comprising Cre recombinase and estrogen receptor type 2 (ER2). Mice expressing the Cre-ER2 fusion protein alone were used as a WT control. In this murine model, the Cre-ER2 fusion is inserted on the Y chromosome (Wirth et al., 2008); therefore, only male mice were used. All mice were housed under control conditions with a 12:12 light/dark cycle at 21°C and 55% humidity, with free access to food and water.

Myh11-Cre^ERT2^ mice were routinely genotyped using the following primer sets: forward 5´-tga-ccc-cat-ctc-ttc-act-cc-3´ and reverse 5´-aac-tcc-acg-acc-acc-tca-tc-3´; yielding a 225 base pair amplicon of WT allele; or 5´-agt-ccc-tca-cat-cct-cag-gtt-3´, yielding a 287 base pair amplicon for the knock-in allele. Floxed TMEM16A mice were genotyped using forward 5´-cca-cat-aag-ggg-cag-tga-gt-3´ and reverse 5´-gct-cca-gct-tcc-agt-gaa-tc-3´ primers, with 947 and 2824 base pair amplicons for the WT and floxed locus, respectively. Mouse strains were regularly refreshed, at least annually, by backcrossing with the background mouse strain C57BL/6JRj (Janvier Labs, Le Genest-Saint-Isle, France).

Homozygous floxed TMEM16A Myh11-Cre^ERT2^ mice were compared with Myh11-Cre^ERT2^ mice following tamoxifen treatment. From the age of 12 weeks, mice were injected for five consecutive days with tamoxifen (20 mg•kg^-1^, *i.p.*) dissolved in corn oil (5 mg•ml^-1^). Mice were used in the experimental protocol a week after the last tamoxifen injection, at the earliest.

Genotypification by Western blotting

Mouse genotype was validated with Western blot for TMEM16A protein expression in smooth muscle cells. Thoracic aortae were dissected from euthanized mice, snap frozen in liquid N_2_, and stored at -70˚C. The samples were lysed, as described previously [1]. A 1x sample buffer was prepared and loaded to stain-free 4–15% gels (CriterionTM TGX Stain-freeTM precast gel, BioRad, Hercules, CA, USA), followed by electrophoresis and transfer onto polyvinylidenfluorid (PVDF) membranes (Merck Millipore, Hellerup, Denmark). The membrane was blocked for 2 hours in Tris-buffered saline (TBS; in mM: 50 Tris, 150 NaCl, pH 7.6) with 0.1% Tween (TBS-T) and 5% non-fat dry milk (Blotting-Grade, Bio-Rad). Afterwards, the membrane was incubated overnight at 4°C in blocking buffer, as above. The TMEM16A-specific band was identified with primary antibody (1:200, ab53212; Cambridge, UK), followed by HRP-conjugated secondary goat-anti-rabbit antibody (1:2000 Cell Signaling Technology) for 2 hours. Band density was analyzed with ImageJ software.

Voltage clamp recordings of Ca^2+^-activated Cl^-^ current in smooth muscle cells

Six WT and five TMEM16A SM-KO brains were dissected in ice-cold physiological salt solution (PSS; in mM: NaCl 115.8, KCl 2.82, KH_2_PO_4_ 1.18, MgSO_4_ 1.2, NaHCO_3_ 25, CaCl_2_ 1.6, EDTA 0.03, glucose 5.5, gassed with 5 % CO_2_ in air and adjusted to pH 7.4) and middle cerebral arteries with first two order branches were isolated for subsequent papain digestion [3, 4]. Arterial segments were stored 4˚C overnight in solution for enzymatic digestion that contained (in mM): NaCl 110, KCl 5, MgCl_2_ 2, KH_2_PO_4_ 0.5, NaH_2_PO_4_ 0.5, NaHCO_3_ 10, CaCl_2_ 0.16, EDTA 0.49, Na-HEPES 10, glucose 10, taurine 10 at pH 7.0, as well as 1.5 mg•ml^−1^ papain, 1.6 mg•ml^−1^ albumin, and 0.4 mg•ml^−1^ dl-dithiothreitol. The next day, arterial segments in this enzymatic solution were incubated for approximately 5 min at 37˚C, and then, arterial segments were transferred into extracellular solution (in mM: CsCl 140, CaCl_2_ 0.1, HEPES 10 at pH 7.4) for smooth muscle cell isolation by gentle trituration with a pipette. Cells were allowed to attach at the bottom of a plastic Petri dish for 15 min, then washed once with the extracellular solution and used for 2 hours of patch clamp recordings.

All experiments were made at room temperature (22–24°C). Patch pipettes were prepared from borosilicate glass (PG15OT-7.5; Harvard Apparatus) pulled on a P-97 puller (Sutter Instrument Co.) and fire-polished to achieve tip resistances of approximately 5±2 MΩ. Pipettes were backfilled with solution containing (in mM): CsCl 140, Ca(OH)_2_ 5.5, MgATP 0.1, EGTA 6, HEPES 10, at pH 7.35, where free intracellular Ca^2+^ was estimated using WEBMAXC v. 2.22 (Chris Patton, Stanford University, CA, USA), approximately 900 nM [4, 5]. Recordings were made in whole-cell configuration using Axopatch 200B amplifier (Axon Instruments, Inc.) and Clampex 9 software for Windows (Axon Instruments, Inc.). Data were sampled at 2 kHz and filtered at 1 kHz. Series resistance and capacitive current were routinely compensated.

The current-voltage relation was constructed by using a voltage-step protocol, where membrane voltages were stepped from −60 to +60 mV with increments of 10 mV for 500 ms duration each. These conditions were previously shown to induce maximal conventional Ca^2+^-activated Cl^-^ current in smooth muscle cells [6]. The voltage-step protocol was then repeated 10 minutes after superfusion with 10 µM Ani9, a selective inhibitor of the TMEM16A-dependent Ca^2+^-activated Cl^-^ channel [7]. Membrane currents at the last 100 ms of each voltage increment were analyzed using LabChart Pro (v8.1.30, ADInstruments, Sydney, Australia) to construct the current-voltage relationships.

Temporal transient middle cerebral artery occlusion

Eight WT and eight TMEM16A SM-KO mice were exposed to a transient ischemia-reperfusion intervention. Thirty minutes prior to anesthesia and approximately 5 hours after surgery, mice were *i.p.* injected with buprenorphine (0.1 mg•kg^-1^; Temgesic, Indivior Europe Limited, Dublin, Ireland). Furthermore, buprenorphine (7.5 µg•ml^-1^) was administered in drinking water. The mice were anesthetized with isoflurane in 100% O_2_ (3% induction, 1.5-2% maintenance). The depth of anesthesia was assessed by respiratory rate and the loss of the withdrawal reflex to toe pinch. Core body temperature was maintained at 36.5-37.0°C using a homeothermic blanket system (50-7222F, Harvard Apparatus, MA, USA). Ophthalmic ointment (Optha A/S, Gentofte, Denmark) was applied to prevent corneal drying.

Prior to the occlusion, a headbar was attached to the cranium over the contralateral hemisphere. Anesthetized mice lying prone were fixed in a stereotaxic frame, and the fur was removed with depilatory cream (Veet, Reckitt Benckiser Group PLC, Slough, UK). The skin and periosteum on the head were removed, and the tissue border was sealed with surgical cyanoacrylate adhesive (VetBond, 3M, St. Paul, MN, USA). A head bar was attached to the dry, clean skull surface using cyanoacrylate adhesive (Loctite, Henkel, Düsseldorf, Germany), followed by a thin layer of self-curing dental cement (Meliodent Rapid Repair, Kulzer, Sweden). To optimize the optical assessment of the brain [8, 9], 3-mm cover glass (CG00E1, ThorLabs Inc., Newton, NJ, USA) was applied on the intact skull with warm solution of 2% agarose (Sigma-Aldrich, Denmark) in between, and secured at the edges with a thin layer of cyanoacrylate.

A transient middle cerebral artery occlusion (MCAO) was induced as previously described, with minor modifications [10]. Mice were prone and fixed using the head bar. An incision of the right temporal muscle was made, and the branches of the MCA were inspected through the skull. A 2-mm craniectomy was made in the temporal bone over the distal MCA, keeping the dura intact. To initiate ischemia, a compression pipette was constructed using two fused blunted borosilicate glass micropipettes [10]. The pipette was positioned perpendicular to the exposed MCA with a micromanipulator and gently advanced to compress the artery trunk. Following 1 hour of stable compression, the pipette was retracted to initiate reperfusion for 24 hours, and then, mice were euthanized with cervical dislocation. The stability of occlusion was observed with Laser Speckle Contrast Imaging. Mice exhibiting a Blood Flow Index (BFI) reduction larger than 50% in the parenchyma were included in the downstream analysis.

In vivo blood flow index measurements by Laser Speckle Contrast Imaging

Dynamic cerebral blood flow in the ipsilateral hemisphere was assessed using Laser Speckle Contrast Imaging [8, 9, 11] using a 1000 × 1000 pixels field of view, with a framerate of 100 frames s^-1^ at baseline, during the temporal MCAO, immediately after pipette release, and again 24 hours after pipette retraction. Imaging was performed on isoflurane (1.5% in 100% O_2_) anesthetized mice with spontaneous respiration. Core body temperature was maintained at 36.5-37.0°C using a homeothermic blanket system (50-7222F, Harvard Apparatus, MA, USA).

To achieve reliable and repeatable measurements with LSCI, we ensured that the imaging parameters were in an optimal range: the speckle-to-pixel size ratio was maintained at approximately 2, and a stabilized laser diode with long coherence length was used. In detail, a near-infrared light was delivered to the cranial surface using a volume-holographic-gating stabilized laser diode (785 nm, FVP785M), controlled by the diode driver CLD1011LP (Thorlabs Inc., Newton, NJ, USA) [12]. Furthermore, a linear polarizer in cross-polarization configuration was installed [9, 12, 13]. Backscattered light was collected by the imaging lens (VZM 200i, Edmund Optics, Barrington, NJ, USA) mounted on the video lens (VZM 200i, Edmund Optics, Barrington, NJ, USA) and recorded with a CMOS camera (acA2000-165umNIR; Basler AG, Ahrensburg, Germany). The parameters were maintained constant, and no changes to the system were introduced during the study. These steps ensured that LSCI measurements were compatible longitudinally and across animal groups.

Furthermore, during the analysis step, potential scattering artifacts in the BFI measurements were minimized by performing temporal contrast analysis over 25 consecutive frames, which is less sensitive to static scattering [14]. Data were analyzed in MATLAB software (v. 2013b; MathWorks, Natick, MA, USA). As all imaging system parameters were kept constant throughout the study, the absolute BFI values across recordings were comparable [9, 14, 15].

Cylindrical behavioral test

Behavioral difference between genotypes and sensorimotor deficits after ischemia-reperfusion intervention were assessed with a cylinder test. Mice were tested at baseline and at 24±2 hours after ischemia-reperfusion. The mice were placed in a cylinder under standardized conditions, with one person only handling, using the same room, light, and temperature settings, and with no human interaction for 10 minutes, while a video recording was made from above.

Infarct validation with TTC staining

The outcome of a transient ischemia-reperfusion intervention was assessed with 2,3,5-triphenyltetrazolium chloride (TTC) staining. After euthanasia, brains were dissected and coronally sliced into 2-mm sections. Brain slices were then incubated at 37˚C in 2.0% TTC (Sigma-Aldrich, Denmark) for 8-10 minutes and washed 3-times in PBS. Post-stained imaging of the brain slices was performed following 1 hour of fixation in 4% formaldehyde, using Epson Perfection V700 Photo scanner (Epson Corporation, Tokyo, Japan).

Pericyte staining

Brain slices used for TTC staining were embedded in paraffin and sectioned in 5 μm slices at the level of the infarct core. Sections were incubated overnight with primary antibody (1:500 rabbit anti‐PDGFRβ, #ab32570, Abcam, Cambridge, UK) at 4 °C, followed by 2 hours incubation with matching secondary antibody (1:500 goat anti‐rabbit IgG, Alexa Fluor 488, #ab150077, Abcam) at 21 °C. Autofluorescence was reduced by 60-second incubation with TrueBlack Autofluorescence Quencher (1:20, Biotium, Fremont, CA). Before mounting, samples were stained with lectin at 21 °C (1:100 Lycopersicon Esculentum [Tomato] Lectin, DyLight 649, DL‐1178‐1 [10 μg/mL], Vector Labs, Newark, CA) to visualize the vasculature and with 300 nM 4′, 6‐diamidino‐2‐phenylindole (DAPI) for nuclei staining *[16]*. The sections were imaged using Olympus VS120 slide scanner (Olympus, Tokyo, Japan) with ×40 air objective (NA 0.95) and resolution 0.17 µm/pixel.

Immunohistochemistry

Two WT and two TMEM16A SM-KO mice were euthanized, and their brains were dissected in phosphate-buffered saline (PBS; in mM: NaCl 137, KCl 2.7, Na_2_HPO_4_ 8.2, KH_2_PO_4_ 1.8, at pH 7.4) at 4°C, following immediate 24 hours emersion in 4% paraformaldehyde (PFA) in PBS. Then, brains were embedded in paraffin and sliced in 10 µm thick coronal slices. Deparaffinized slices were permeabilized with 0.2% Triton X-100 and blocked after washing in PBS with 5% fetal bovine serum and 1% bovine serum albumin (all from Sigma Aldrich, Søborg, Denmark). Slices were incubated overnight at 4°C with primarily antibodies against TMEM16A (1:500; catalog #ab323181, Abcam, Cambridge, UK) and α-smooth-muscle-actin (1:200; catalog #ab112022, Abcam, Cambridge, UK), washed next day in PBS and stained 2 hours at 4°C in dark with secondary antibodies (1:1000; donkey anti-rabbit IgG Alexa Fluor 568 (catalog #A10042) for TMEM16A and donkey anti-goat IgG Alexa Fluor 647 (catalog # A-21447); Thermo Fisher Scientific, Denmark). After washing in PBS, cover glass was mounted on fluromount with DAPI (catalog #00-4959-52; Thermo Fisher Scientific, Denmark). Background fluorescence was assessed by staining the coronal brain slices with secondary antibodies only (2º antibodies). Imaging was conducted using the Olympus VS120 slide scanner (Olympus, Tokyo, Japan) at x40 magnification and examined with QuPath, an open-source software for digital image analysis [17].

Proteomics

Cortices from brain slices stained with TTC were used for proteome analysis. Peptides for tandem mass tagging were prepared by incubating samples in 8M urea in the presence of 0.5 µg lys-C for 4 hours at 30°C. This was initiated with water sonication in three 5-minute cycles followed by dilution to 1 M urea by tetraethylammonium bicarbonate (TEAB) and incubation overnight at 30° C after addition of 1 µg trypsin. Next day, peptide samples were labelled with tandem mass tags from a 10-plex Tandem Mass Tags (TMT): 127N, 127C, 128N, 128C, 129N, 129C, 130N, 130C, and 131. A pool of all samples was labelled with mass tag 126 and served as a reference channel. Tagged peptide samples were mixed in two sets of tagged peptide mixture and analyzed by reversed phase nano-liquid chromatography tandem mass spectrometry (RP-nanoLC-MS/MS) *[18]*. All raw data files were processed using the Proteome Discoverer software (v. 2.4.0.305) and searched with the MSPepSearch and the Sequest HT search algorithm. The search parameters for the MSPepSearch were kept at default except the precursor and fragment tolerances, which were set to 15 ppm. The TMT-specific spectral library was prepared by Shen et al. [19] and imported into Proteome Discoverer. Sequest HT search parameters were set to default except for MS accuracy of 8 ppm, MSMS accuracy of 0.05 Da for HCD data, with two missed cleavages allowed. Fixed modifications were set to carbamidomethylation at cysteine residues, TMT 6-plex N-terminal, and TMT 6-plex on lysine residues. Variable modifications were set to methionine oxidation, deamidation of asparagine and glutamine, and N-terminal acetylation. Sequest HT searches were performed against the Uniprot mouse database (25252 entries, downloaded on 30th September 2019).

Pressure myography

After euthanasia, brains were dissected into ice-cold PSS and middle cerebral artery segments were dissected to be cannulated from both ends with glass microcannulas in a pressure myograph (111P, DMT) [8, 20]. Arterial segments were equilibrated for 30 minutes at 37˚C in bio-air (5% CO_2_ in air) aerated PSS at 60 mmHg transmural pressure. Then, transmural pressure was elevated to 120 mmHg and the arterial segment stretched to be aligned. The pressure steps between 40 and 120 mmHg with 20 mmHg increments, 5 minutes each were repeated under control conditions in PSS and after 15 min incubation in Ca^2+^-free PSS in the presence of 10 µM nifedipine and 10 µM Y27632. The outer diameters were measured and degree of active tone at each pressure step was quantified. The Ca^2+^-free PSS composition was similar with PSS, but CaCl_2_ was omitted.

Radiotelemetry

Five WT and eight TMEM16A SM-KO mice at the age of 12-16 weeks were used for radiotelemetry studies. Blood pressure, heart rate, and activity were measured in freely moving mice instrumented with radiotelemetric probes. Ten minutes prior to anesthesia, mice were s.c. injected with buprenorphine (1 mg•kg^-1^). Mice were anesthetized with isoflurane (3% induction, 1.5-2% maintenance in 100% O_2_), their body temperature was maintained at 37°C with a thermostatically controlled warming platform. A midline incision through the shaved skin on the neck was made to access the carotid artery. The catheter of a PA-C10 radiotelemetry transmitter (Data Sciences International) was placed into the left carotid artery, and the transmitter body was placed in a subcutaneous pocket. The catheter was fixed to the artery using 6-0 silk sutures, and the skin incision was closed with 6-0 monofilament thread. Buprenorphine (7.5 µg•ml^-1^) was administered in drinking water for the following three days. Mice were allowed to recover for at least one week before measurements were started. Telemetry signals were recorded at 256 Hz with Ponemah 8 (Data Sciences International). The signal was averaged per hour.

Data analysis and statistics

To avoid genotype bias, all procedures and analyses have been performed in a randomized manner by blinded researchers.

Current-voltage relations were fitted with a fifth-order polynomial equation (Y=B_0_ + B_1_•X + B_2_•X^2^ + B_3_•X^3^ + B_4_•X^4^ + B_5_•X^5^), whose parameters were used to compare data sets using the extra sum-of-squares *F* test. Circadian dynamics of radiotelemetry parameters were fitted in the cosinor function: Y=mesor+amplitude•cos(period•(X-acrophase), which parameters were used for an extra sum-of-squares *F* test comparison of data sets.

BFI analysis was performed by placing a region of interest (ROI) in different regions of a cortical image, including the middle cerebral artery branches, larger veins at different grades, anterior cerebral artery branches, and brain parenchyma. Individually, the BFI for each ROI was weighted by pixel size. The weighted BFI was then categorized into ‘2^nd^ and 3^rd^ MCA’, ‘Larger downstream MCA branches’, ‘Parenchyma’, ‘Smaller veins’, and ‘Larger veins’. Only mice exhibiting a BFI reduction larger than 50% in the parenchyma in response to middle cerebral artery occlusion were included in the downstream analysis.

Behavioral analysis of video recordings was performed with Ethovision software (ver. 11, Noldus Information Technology, Netherlands) to assess travel distance, velocity, rearing, and grooming. An observer also determined the amount of time that the right, left, or both paws were in contact with the cylinder walls. Paw-use asymmetry was calculated as (% left paw use – % right paw use)/(% left paw use + % right paw use) [9, 21, 22]. Pearson’s correlation coefficient, r, was used to assess the correlation between the large arterial BFI and behavior in the respective genotype at baseline and following 24h of reperfusion.

Hemisphere and infarct size were measured using ImageJ software (ver. 1.54p, National Institutes of Health, USA) and calculated as volume for 3 consecutive slices over cerebrum in Microsoft Excel (Microsoft 365, Redmond, WA, USA). The infarct volume was calculated as the percentage of the total hemisphere volume. Volume changes in the ipsilateral hemisphere were assessed in relation to the contralateral hemisphere volume.

Images of pericyte staining were processed with QuPath (ver. 0.5.1) and ImageJ software (ver. 1.54f, National Institutes of Health) using Enhance Image macro. Capillary diameter was assessed automatically in ImageJ (National Institutes of Health) using VasoMetrics macro. An association of lectin-labeled capillaries with a pericyte body was based on colocalization of PDGFRβ. The diameter measurements were done for a 10 µm distance at a1 µm interval along the capillary length in both directions from the pericyte soma. The measurements were repeated at least 5 times for different capillaries and averaged per slide. Capillary density (the percentage of vessel area inside the image area) and lacunarity (a measure of how non-uniform a vascular network; has no units) were assessed with AngioTool64 in accordance with software guidelines (ver. 0.6a; National Cancer Institute, USA). The position-matched regions of interest were selected within the ipsilateral and contralateral cortical territory supplied by the middle cerebral artery.

Realtive protein abundance identified with proteomics was compared between the groups using unpaired *t*-test and between hemispheres using paired *t*-test, and the changes were expressed as a fold change between compared groups (Suppl. Table 2). These results were uploaded to IPA (IPA software, Qiagen, Redwood City, CA, USA) for further interpretation. The gene ontology pathways were analyzed for enrichment of differentially abundant proteins, and their associations with the changes in ingenuity canonical pathways were suggested.

A list of differentially abundant proteins in bulk astrocyte lysates from SoCS2-/- and wild type mice of P8 and 2 months of age was uploaded to IPA for functional interpretation. First, gene ontology pathways were analyzed for enrichment of differentially abundant proteins. A negative z-score indicates suppression of the pathway, while a positive z-score indicates enhancement. The list of quantified proteins was uploaded into IPA software (Qiagen, Redwood City, CA, USA) for further analysis, where the proteins, whose abundance differed significantly between genotypes, were identified, and their association with the changes in ingenuity canonical pathways was suggested.

The statistical analysis was performed in Prism ver. 10.6.1 (GraphPad Software, Boston, MA, USA). Data were compared using unpaired or paired *t*-tests, one-way and two-way ANOVA followed by correction for multiple comparisons, where appropriate. The type of statistics used is specifically indicated in the figure legends. All data are represented as mean ± Standard Deviation (SD). An asterisk (*) indicates analysis within the WT group, an octothorpe (#) indicates the intervention effect within the TMEM16A SM-KO group, whereas a double dagger (‡) indicates the comparisons between WT and TMEM16A SM-KO at the same condition or timepoint. *P*-values below 0.05 are considered significant, with the number of symbols indicating the degree of significance.

Supplementary figures


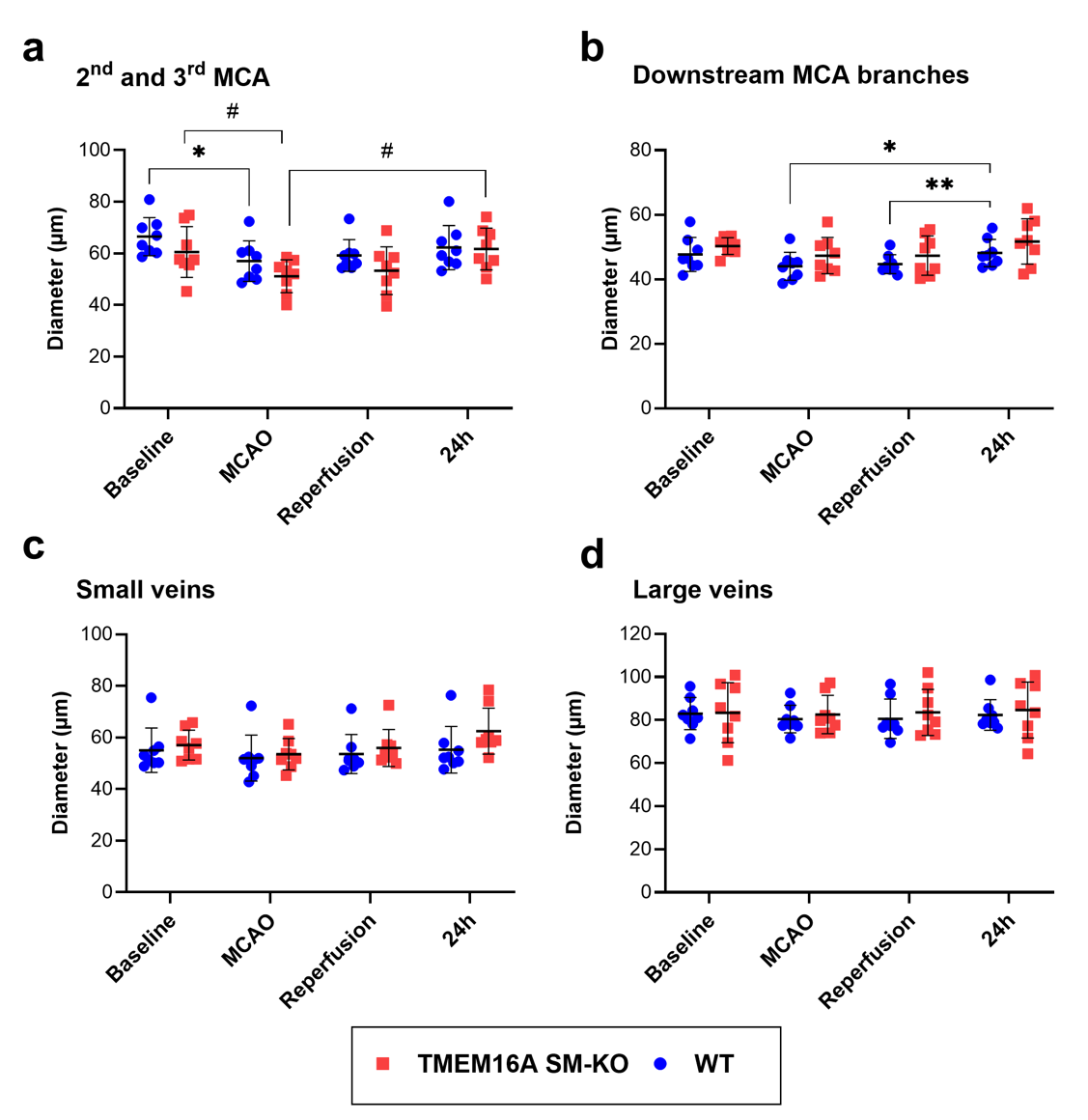


**Supplementary Figure 1. Vessel diameter changes during middle cerebral artery occlusion-reperfusion (MCAO) do not differ between WT and TMEM16A SM-KO mice.** Diameters of 2^nd^- and 3^rd^- order branches of middle cerebral artery (MCA) (a), small branches of MCA downstream for occlusion (b), small (c), and large draining veins (d) were measured throughout the experimental protocol at baseline, during MCAO, at the onset of reperfusion, and 24 hours after reperfusion. See Fig. 3a for different vascular segment identification. *, ** indicates *P* < 0.05 and < 0.01 for intervention effect within WT group. # indicates *P* < 0.05 for intervention effect within TMEM16A SM-KO group. Data were compared with two-way ANOVA followed by Tukey’s correction for multiple comparisons. *n* = 8 per group.


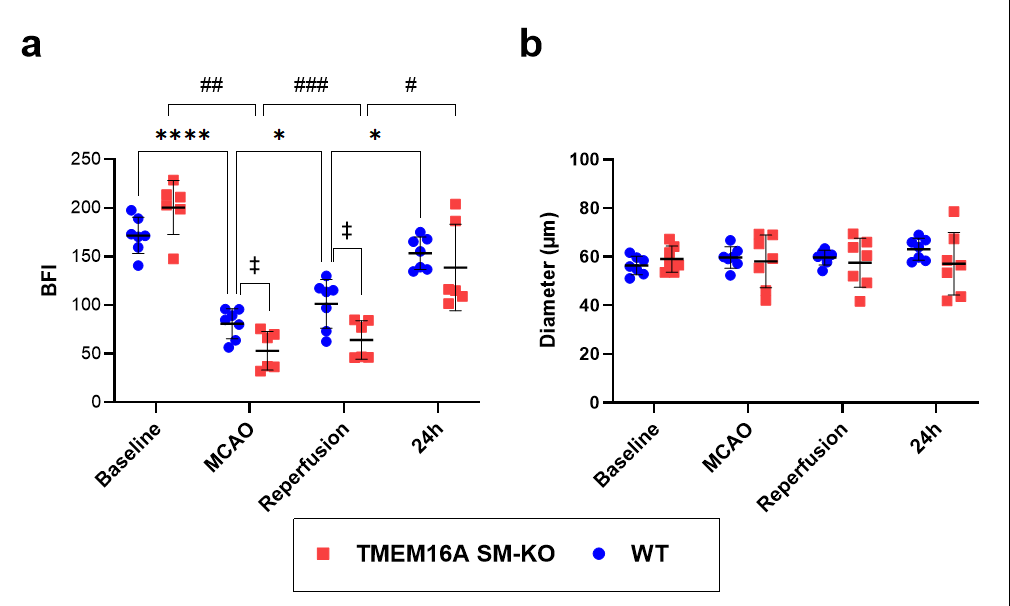


**Supplementary Figure 2. Occlusion of middle cerebral artery reduced** **anterior cerebral artery blood flow, with a greater reduction in TMEM16A SM-KO mice than in WT mice.** Weighted BFI (a) and diameter (b) in the branches of anterior cerebral artery, as indicated Fig. 3a (black arrows). Measurements were performed throughout the experimental protocol at baseline, during middle cerebral artery occlusion (MACO), at the onset of reperfusion, and 24 hours of reperfusion. *, **, and **** indicate *P* < 0.05, < 0.01, and < 0.0001 for the effect within WT group. #, ##, and ### for *P* < 0.05, 0.01, and < 0.001 for the effect of intervention within TMEM16A SM-KO group. ‡ indicates *P* < 0.05 for comparisons between WT and TMEM16A SM-KO at the same timepoint. Data were compared using a two-way ANOVA followed by Tukey's post hoc test for multiple comparisons. *n* = 6-7.


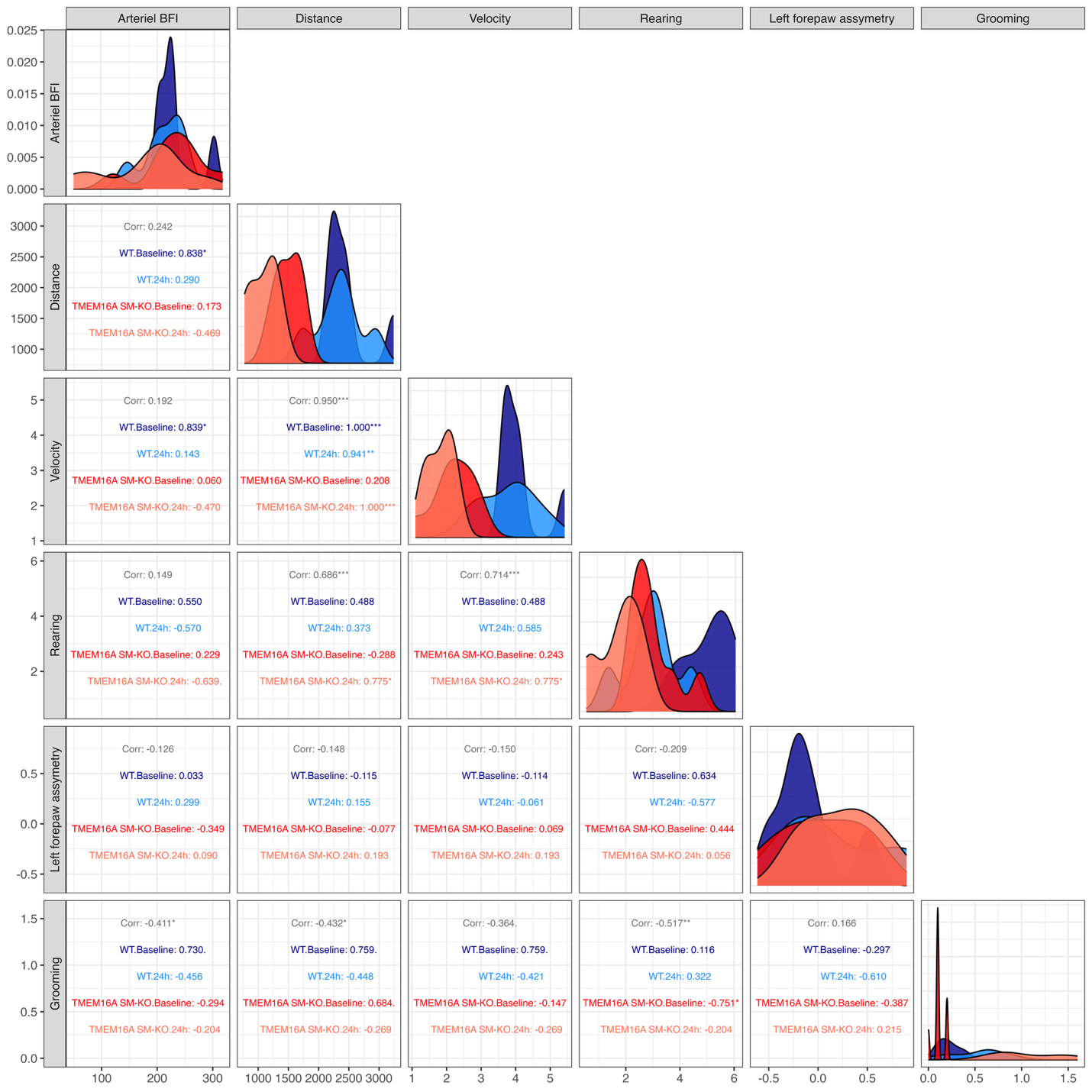


**Supplementary Figure 3. Correlation analyses between arterial BFI and locomotor parameters revealed heterogeneous associations across behavioral measures, genotypes, and time points.** Pearson’s correlation matrix of arterial blood flow index (BFI) in relation to behavioral changes at baseline and following 24h of reperfusion in WT (blue, *n =* 6) and TMEM16A SM-KO (red, *n =* 8) mice. Grey indicates correlations calculated across all samples, regardless of genotype and timeline. *, ** and *** indicate *P* < 0.05, < 0.01 and < 0.001, respectively.


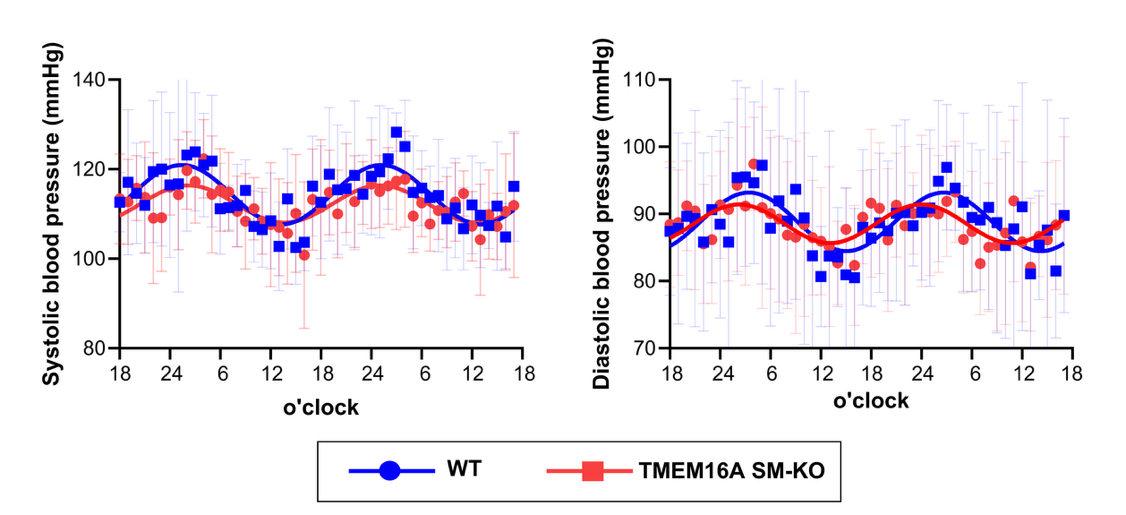


**Supplementary Figure 4. Blood pressure parameters do not differ between TMEM16A SM-KO and WT mice.** Systolic and diastolic arterial pressure were recorded radiotelemetrically over 48 hours in TMEM16A SM-KO (red; *n* = 8) and WT (blue; *n* = 5) mice. Both parameters exhibited circadian variations, with no difference between genotypes. See Fig. 7a,b.


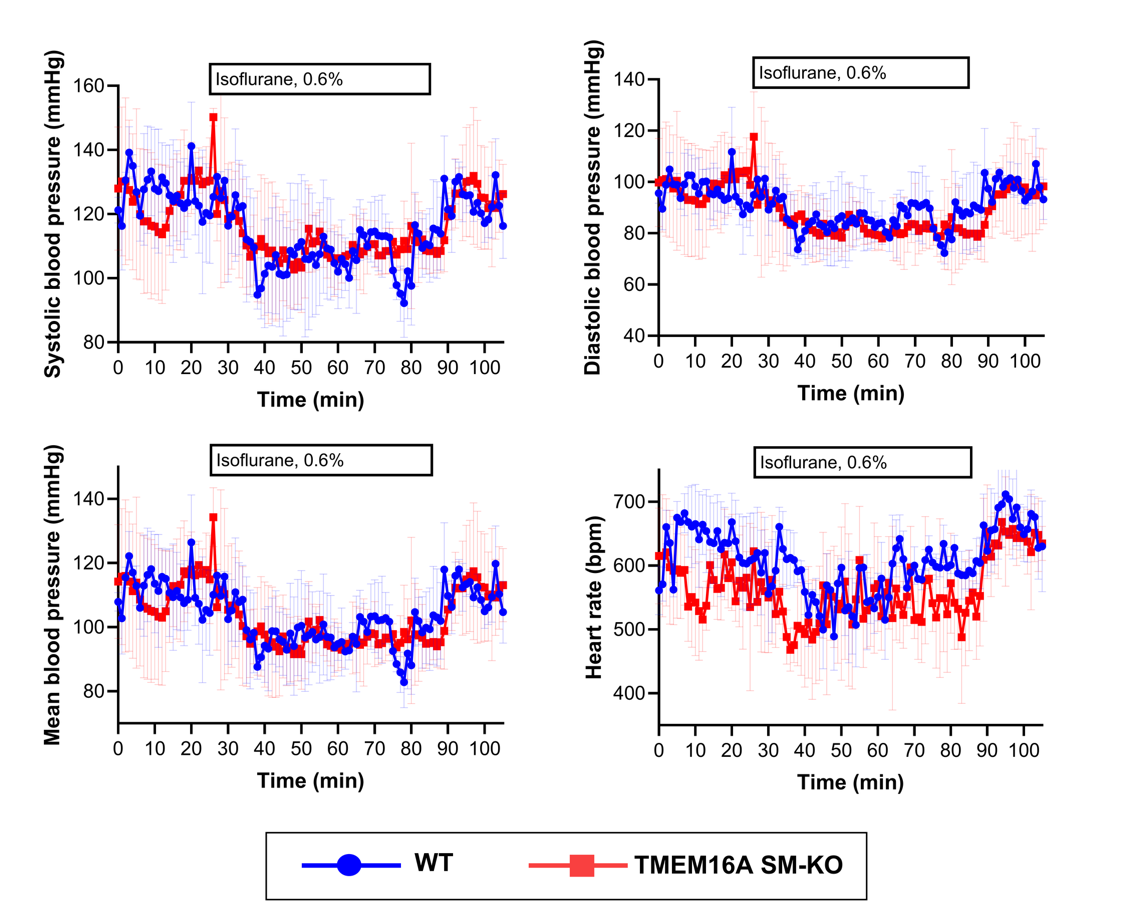


**Supplementary Figure 5. Cardiovascular response to mild isoflurane (0.6%) anesthesia do not differ between TMEM16A SM-KO and WT mice.** Systolic, diastolic, and mean arterial pressure, as well as heart rate were measured in TMEM16A SM-KO (*n* = 4) and WT (*n* = 4) mice under control conditions, during 1 hour of 0.6% isoflurane anesthesia, and during recovery from anesthesia.


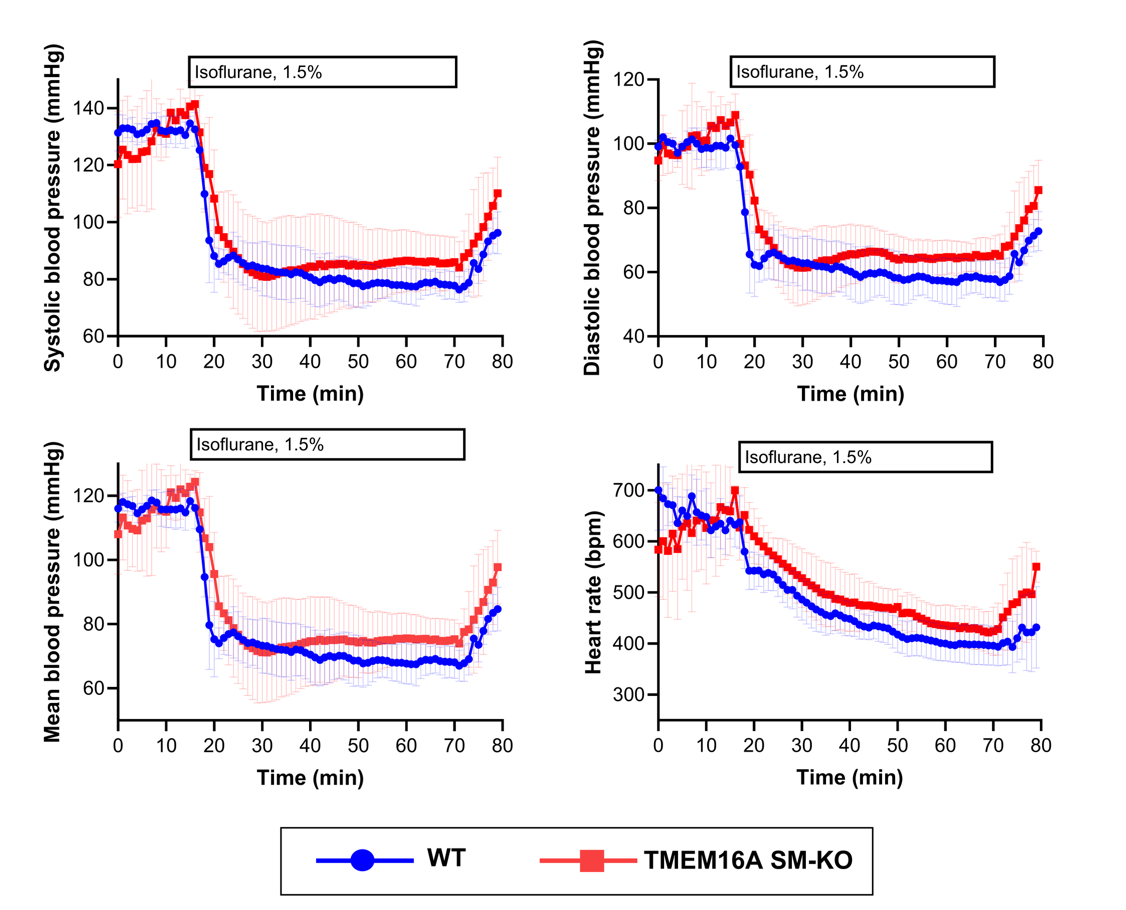


**Supplementary Figure 6. Cardiovascular responses to isoflurane (1.5%) anesthesia do not differ between TMEM16A SM-KO and WT mice.** Systolic, diastolic, and mean arterial pressure, as well as heart rate were measured in TMEM16A SM-KO (*n* = 4) and WT (*n* = 4) mice under control conditions, during 1 hour of 1.5% isoflurane anesthesia, and during recovery. See also Fig. 7d-f.


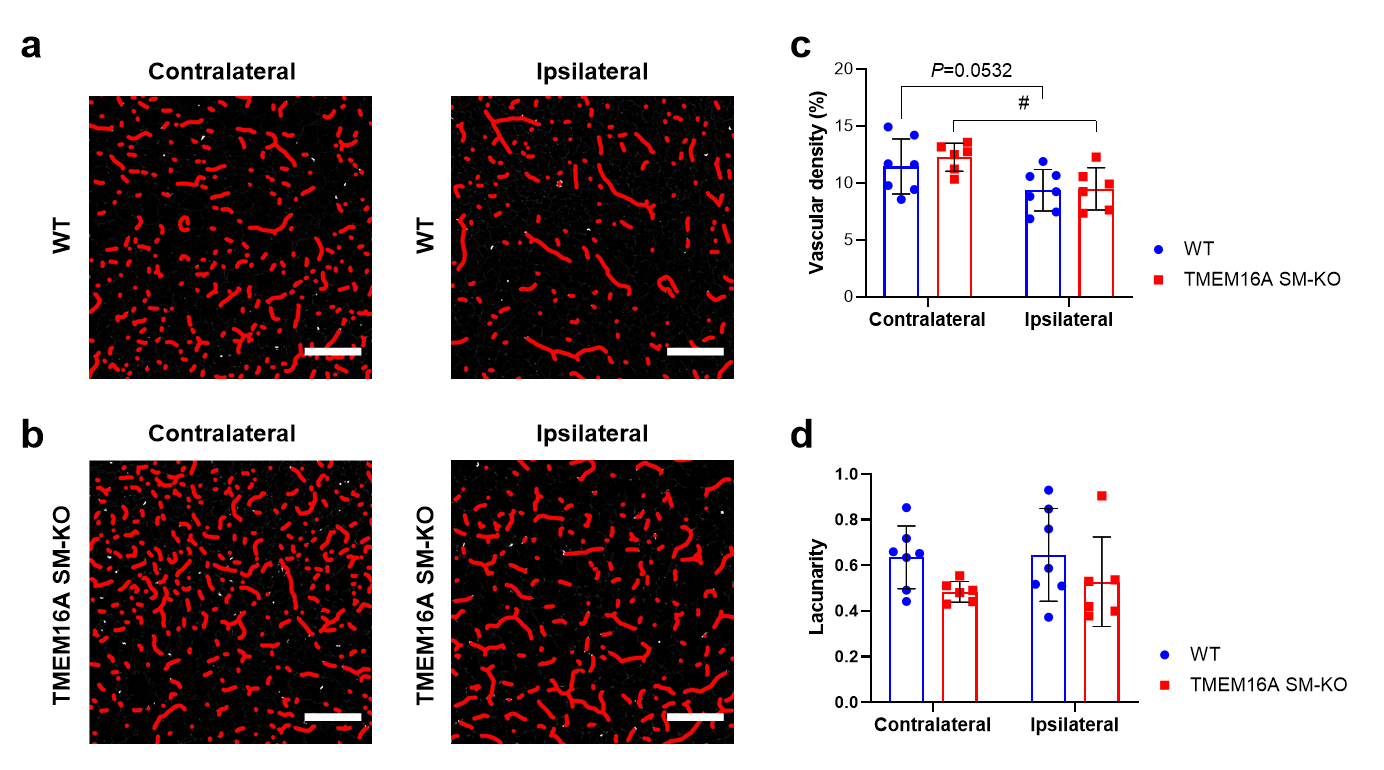


**Supplementary Figure 7. Vascularization does not differ between WT and TMEM16A SM-KO murine brains**. AngioTools analysis of ipsilateral and position-matched contralateral cortices from WT (*n* = 7) and TMEM16A SM-KO (*n* = 7) mice (a and b, representative images). Regions of interest were selected within the cortical territory supplied by the middle cerebral artery [23]. Vascular density was not different between genotypes at the corresponding cortical locations; however, vascular density was decreased in the ipsilateral hemisphere in comparison with the contralateral hemisphere in both genotypes (c). No difference in lacunarity was detected among the groups. Scale bars correspond to 200 µm. # indicates *P* < 0.05 for interventions within the TMEM16A SM-KO group; two-way ANOVA followed by post-hoc Fisher’s least significant difference test.


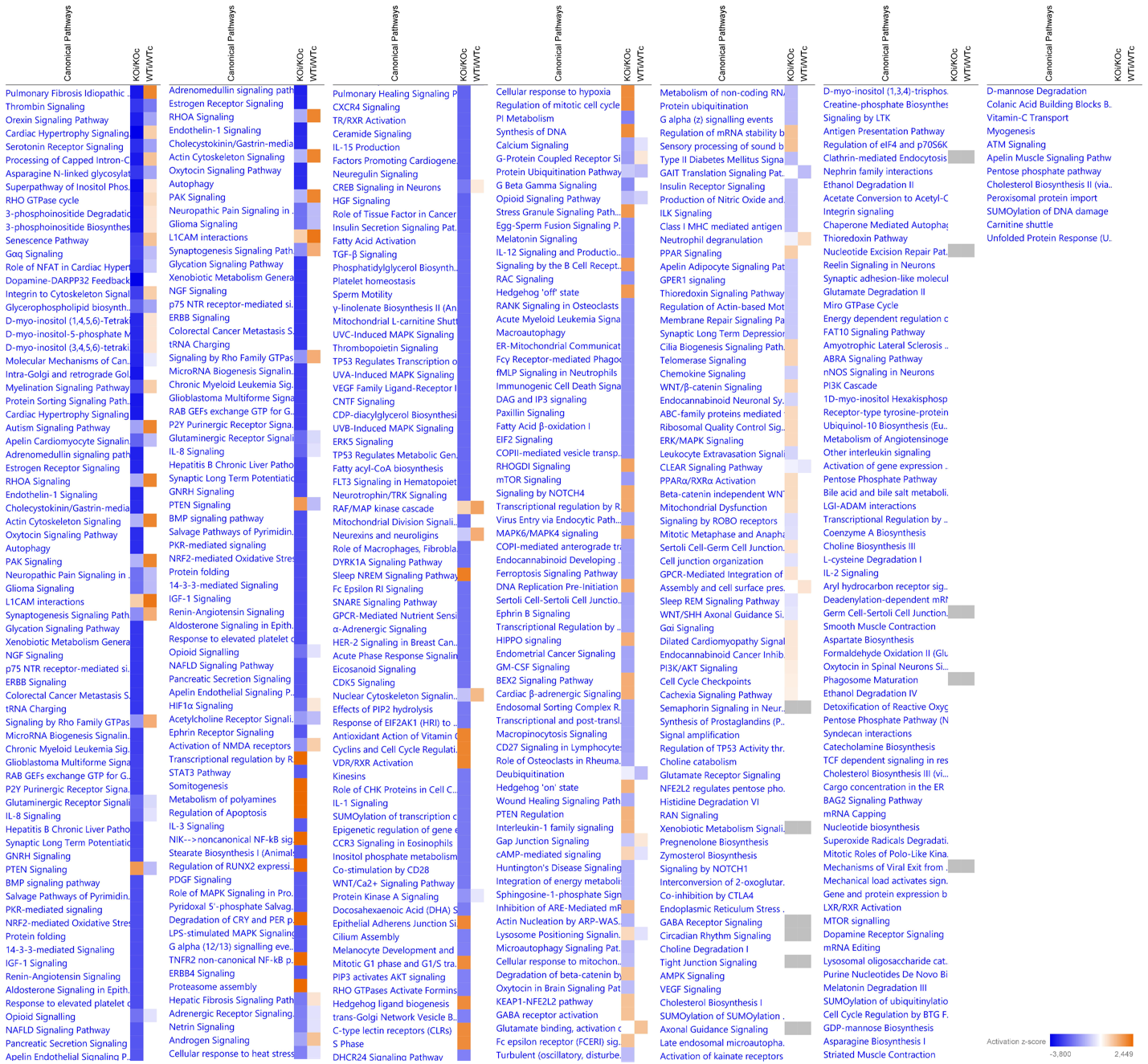


**Supplementary Figure 8. Proteomic pathway analysis reveals enhanced ischemia-reperfusion-associated signaling changes in TMEM16A SM-KO cortices compared with WT.** Canonical pathways predicted to be altered in the ipsilateral cortices vs. the contralateral cortices were compared between TMEM16A SM-KO (*n =* 5) and WT (*n =* 4) mice, revealing a greater magnitude of pathway modulation in TMEM16A SM-KO mice. See also Supplementary Table 3 and Fig. 10f.

Supplementary tables

**Supplementary Table 1.** Proteomics data for ipsilateral and contralateral cortices from WT (*n =* 4) and TMEM16A SM-KO mice (*n =* 5). ID of identified proteins, individual expression levels for samples, averaged ratio, and unpaired and paired *t*-test statistics, as appropriate.

**Supplementary Table 2.** Expression analyses for proteomics data for the ipsilateral and contralateral cortices from WT (*n =* 4) and TMEM16A SM-KO mice (*n =* 5). The expression fold changes and corresponding statistics for detected proteins when contralateral cortices or ipsilateral cortices from TMEM16A and WT mice were compared; ipsilateral and contralateral cortices from either WT or TMEM16A mice were compared, as indicated in the Excel file sheets. Dataset generated based on Ingenuity Pathway Analysis, QIAGEN. Downregulated proteins are labeled in blue and upregulated proteins in red. See also Fig. 10.

**Supplementary Table 3**. Ingenuity Canonical Pathways, QIAGEN, suggested to be affected either by TMEM16A SM-KO or by ischemia-reperfusion. Downregulated (blue), upregulated (red) or undefined (black) pathway changes in TMEM16A SM-KO contralateral cortices vs. WT contralateral cortices; or TMEM16A SM-KO ipsilateral cortices vs. WT ipsilateral cortices; or WT ipsilateral vs. WT contralateral cortices; or TMEM16A SM-KO ipsilateral vs. TMEM16A SM-KO contralateral cortices, as indicated in the Excel file sheets. Data suggested by Ingenuity Pathway Analysis, QIAGEN, based on the dataset in Supplementary Table 1.

References

[1] Matchkov VV, Black Joergensen H, Kamaev D, Hoegh Jensen A, Beck HC, Skryabin BV, Aalkjaer C. A paradoxical increase of force development in saphenous and tail arteries from heterozygous ANO1 knockout mice. Physiol Rep 2020;8(22):p.e14645. <https://doi.org/10.14814/phy2.14645>

[2] Wirth A, Benyo Z, Lukasova M, Leutgeb B, Wettschureck N, Gorbey S, Orsy P, Horvath B, Maser-Gluth C, Greiner E, Lemmer B, Schutz G, Gutkind JS, Offermanns S. G12-G13-LARG-mediated signaling in vascular smooth muscle is required for salt-induced hypertension. Nat Med 2008;14(1):pp.64-8. <https://doi.org/10.1038/nm1666>

[3] Schubert R, Krien U, Gagov H. Protons inhibit the BK_Ca_ channel of rat small artery smooth muscle cells. J. Vasc. Res 2001;38(1):pp.30-8. <https://doi.org/> 10.1159/000051027

[4] Matchkov VV, Aalkjaer C, Nilsson H. A cyclic GMP-dependent calcium-activated chloride current in smooth-muscle cells from rat mesenteric resistance arteries. J. Gen. Physiol 2004;123(2):pp.121-34. <https://doi.org/10.1085/jgp.200308972>

[5] Matchkov VV, Aalkjaer C, Nilsson H. Distribution of cGMP-dependent and cGMP-independent Ca^2+^-activated Cl^-^ conductances in smooth muscle cells from different vascular beds and colon. Pflugers Arch 2005;451(2):pp.371-9. <https://doi.org/> 10.1007/s00424-005-1472-9

[6] Matchkov VV, Larsen P, Bouzinova EV, Rojek A, Boedtkjer DM, Golubinskaya V, Pedersen FS, Aalkjaer C, Nilsson H. Bestrophin-3 (vitelliform macular dystrophy 2-like 3 protein) is essential for the cGMP-dependent calcium-activated chloride conductance in vascular smooth muscle cells. Circ Res 2008;103(8):pp.864-72. <https://doi.org/10.1161/CIRCRESAHA.108.178517>

[7] Seo Y, Lee HK, Park J, Jeon DK, Jo S, Jo M, Namkung W. Ani9, A Novel Potent Small-Molecule ANO1 Inhibitor with Negligible Effect on ANO2. PLoS One 2016;11(5):p.e0155771. <https://doi.org/10.1371/journal.pone.0155771>

[8] Staehr C, Hangaard L, Bouzinova EV, Kim S, Rajanathan R, Boegh Jessen P, Luque N, Xie Z, Lykke-Hartmann K, Sandow SL, Aalkjaer C, Matchkov VV. Smooth muscle Ca(2+) sensitization causes hypercontractility of middle cerebral arteries in mice bearing the familial hemiplegic migraine type 2 associated mutation. J Cereb Blood Flow Metab 2018;39(8):pp.1570-87. <https://doi.org/10.1177/0271678X18761712>

[9] Gonzalez Olmos A, Zilpelwar S, Sunil S, Boas DA, Postnov DD. Optimizing the precision of laser speckle contrast imaging. Sci Rep 2023;13(1):p.17970. <https://doi.org/10.1038/s41598-023-45303-z>

[10] Erdener SE, Tang J, Kilic K, Postnov D, Giblin JT, Kura S, Chen IA, Vayisoglu T, Sakadzic S, Schaffer CB, Boas DA. Dynamic capillary stalls in reperfused ischemic penumbra contribute to injury: A hyperacute role for neutrophils in persistent traffic jams. J Cereb Blood Flow Metab 2021;41(2):pp.236-52. <https://doi.org/10.1177/0271678X20914179>

[11] Staehr C, Rajanathan R, Postnov DD, Hangaard L, Bouzinova EV, Lykke-Hartmann K, Bach FW, Sandow SL, Aalkjaer C, Matchkov VV. Abnormal neurovascular coupling as a cause of excess cerebral vasodilation in familial migraine. Cardiovasc Res 2020;116(12):pp.2009-20. <https://doi.org/10.1093/cvr/cvz306>

[12] Postnov DD, Cheng X, Erdener SE, Boas DA. Choosing a laser for laser speckle contrast imaging. Sci Rep 2019;9(1):p.2542. <https://doi.org/10.1038/s41598-019-39137-x>

[13] Akther S, Mikkelsen MB, Postnov DD. Choosing a polarisation configuration for dynamic light scattering and laser speckle contrast imaging. Biomed Opt Express 2024;15(1):pp.336-45. <https://doi.org/10.1364/BOE.507367>

[14] Boas DA, Dunn AK. Laser speckle contrast imaging in biomedical optics. J Biomed Opt 2010;15(1):p.011109. <https://doi.org/10.1117/1.3285504>

[15] Kirkpatrick SJ, Duncan DD, Wells-Gray EM. Detrimental effects of speckle-pixel size matching in laser speckle contrast imaging. Opt Lett 2008;33(24):pp.2886-8. <https://doi.org/10.1364/ol.33.002886>

[16] Staehr C, Giblin JT, Gutierrez-Jimenez E, Guldbrandsen HO, Tang J, Sandow SL, Boas DA, Matchkov VV. Neurovascular Uncoupling Is Linked to Microcirculatory Dysfunction in Regions Outside the Ischemic Core Following Ischemic Stroke. J Am Heart Assoc 2023;12(11):p.e029527. <https://doi.org/10.1161/JAHA.123.029527>

[17] Bankhead P, Loughrey MB, Fernandez JA, Dombrowski Y, McArt DG, Dunne PD, McQuaid S, Gray RT, Murray LJ, Coleman HG, James JA, Salto-Tellez M, Hamilton PW. QuPath: Open source software for digital pathology image analysis. Sci Rep 2017;7(1):p.16878. <https://doi.org/10.1038/s41598-017-17204-5>

[18] Staehr C, Login H, Melnikova EV, Bakun M, Ziemlinska E, Kisiswa L, Ardestani SB, Nolte SS, Beck HC, Hansen LMB, Postnov D, Verkhratsky A, Malik AR, Nykjaer A, Matchkov VV. SorCS2 Is Important for Astrocytic Function in Neurovascular Signaling. Acta Physiol (Oxf) 2025;241(6):p.e70052. <https://doi.org/10.1111/apha.70052>

[19] Shen J, Pagala VR, Breuer AM, Peng J, Bin M, Wang X. Spectral Library Search Improves Assignment of TMT Labeled MS/MS Spectra. J Proteome Res 2018;17(9):pp.3325-31. <https://doi.org/10.1021/acs.jproteome.8b00594>

[20] Matchkov VV, Tarasova OS, Mulvany MJ, Nilsson H. Myogenic response of rat femoral small arteries in relation to wall structure and [Ca^2+^]_i_. Am. J. Physiol 2002;283(1):pp.H118-H25.

[21] Bice AR, Xiao Q, Kong J, Yan P, Rosenthal ZP, Kraft AW, Smith KP, Wieloch T, Lee JM, Culver JP, Bauer AQ. Homotopic contralesional excitation suppresses spontaneous circuit repair and global network reconnections following ischemic stroke. Elife 2022;11. <https://doi.org/10.7554/eLife.68852>

[22] Li CX, Kapoor E, Chen W, Ward LM, Lee DD, Titus A, Reardon KM, Lee JM, Yuede CM, Landsness EC. Manual assessment of cylinder rearing behavior is more sensitive than automated gait evaluations in young, male mice post-stroke of the forepaw somatosensory cortex. J Stroke Cerebrovasc Dis 2025;34(7):p.108325. <https://doi.org/10.1016/j.jstrokecerebrovasdis.2025.108325>

[23] Xiong B, Li A, Lou Y, Chen S, Long B, Peng J, Yang Z, Xu T, Yang X, Li X, Jiang T, Luo Q, Gong H. Precise Cerebral Vascular Atlas in Stereotaxic Coordinates of Whole Mouse Brain. Front Neuroanat 2017;11p.128. <https://doi.org/10.3389/fnana.2017.00128>
