# Supplementary material for: Loss of Vascular TMEM16A Impairs Cerebral Autoregulation and Exacerbates Ischemia–reperfusion Injury
Source: Transl Stroke Res. 2026 Jul 22;17(4):85. doi: 10.1007/s12975-026-01471-4 (PMC13391751; doi:10.1007/s12975-026-01471-4)

Uncropped images for Figure 1a and 1b - *aorta lysate*

Blot stained for TMEM16A

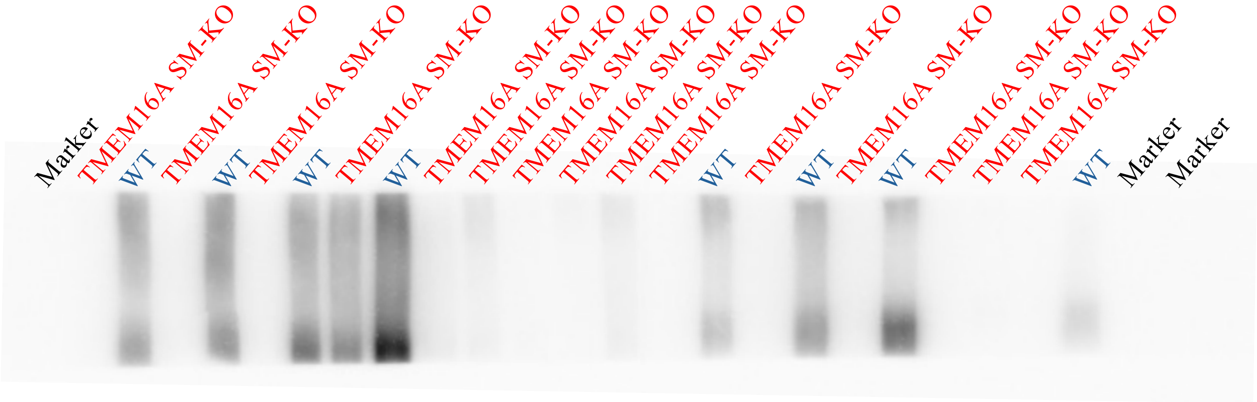

Corresponding molecular weight markers

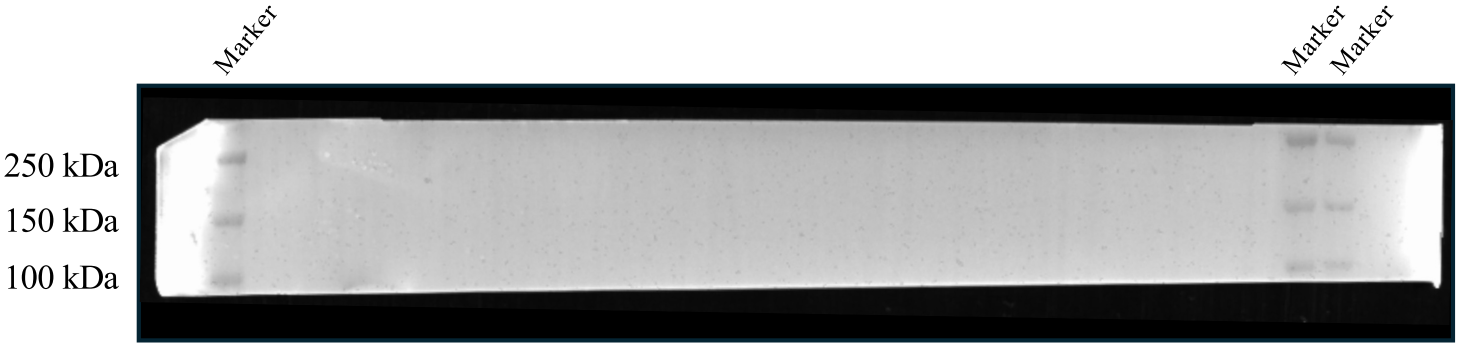

Membrane used for loading normalization

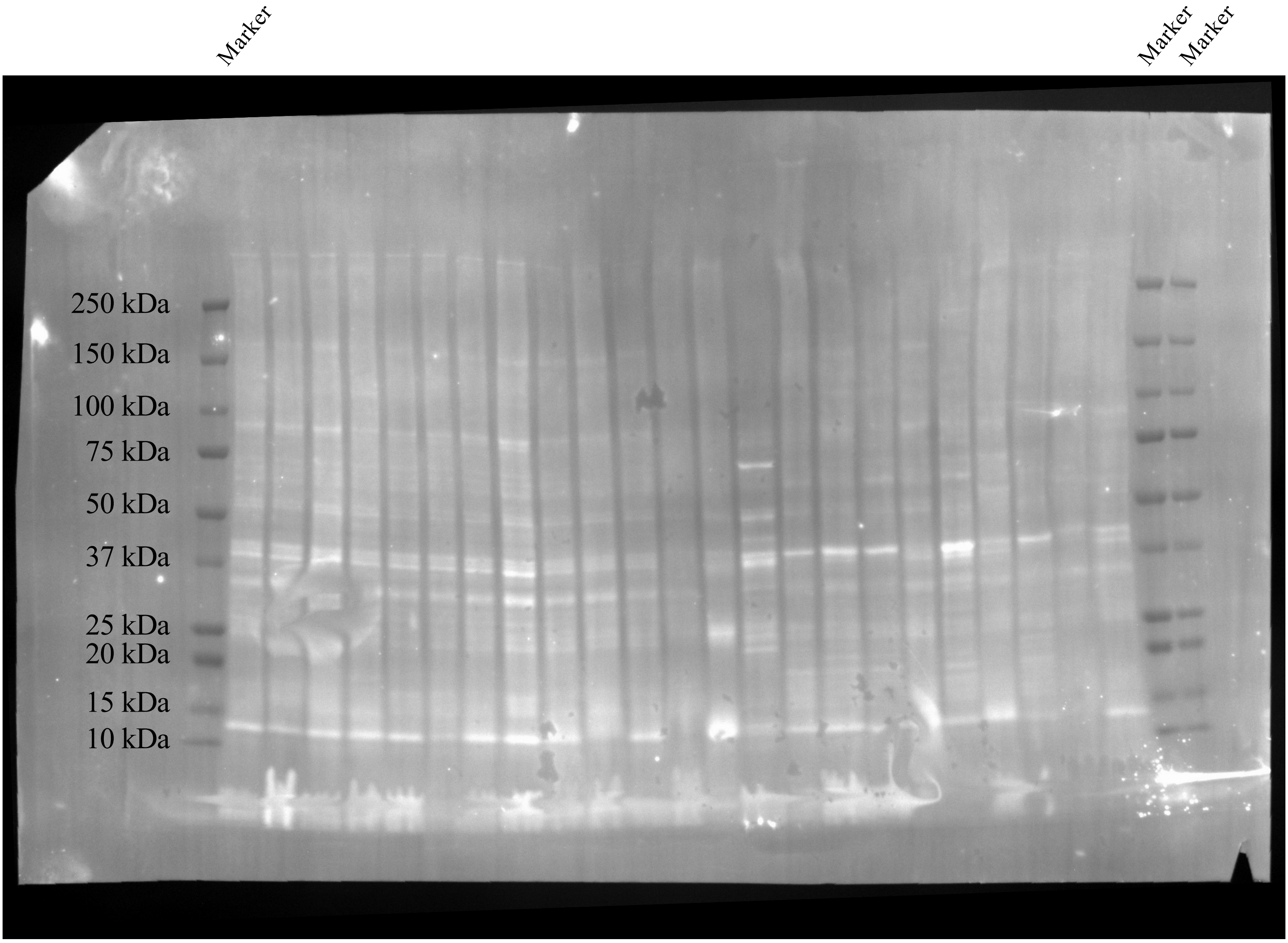

Uncropped images for Figure 1a and 1b (*continuation*) - *aorta lysate*

Blot stained for TMEM16A

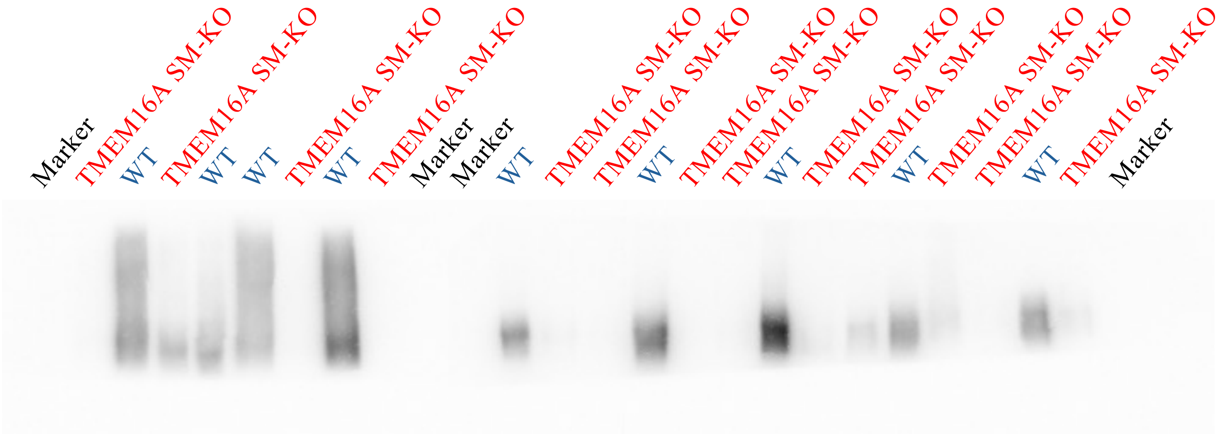

Corresponding molecular weight markers

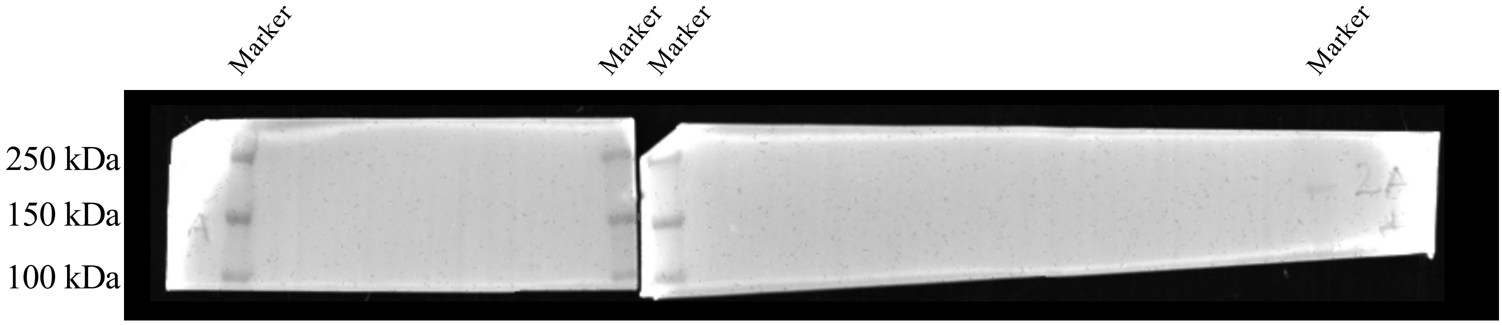

Membrane used for loading normalization

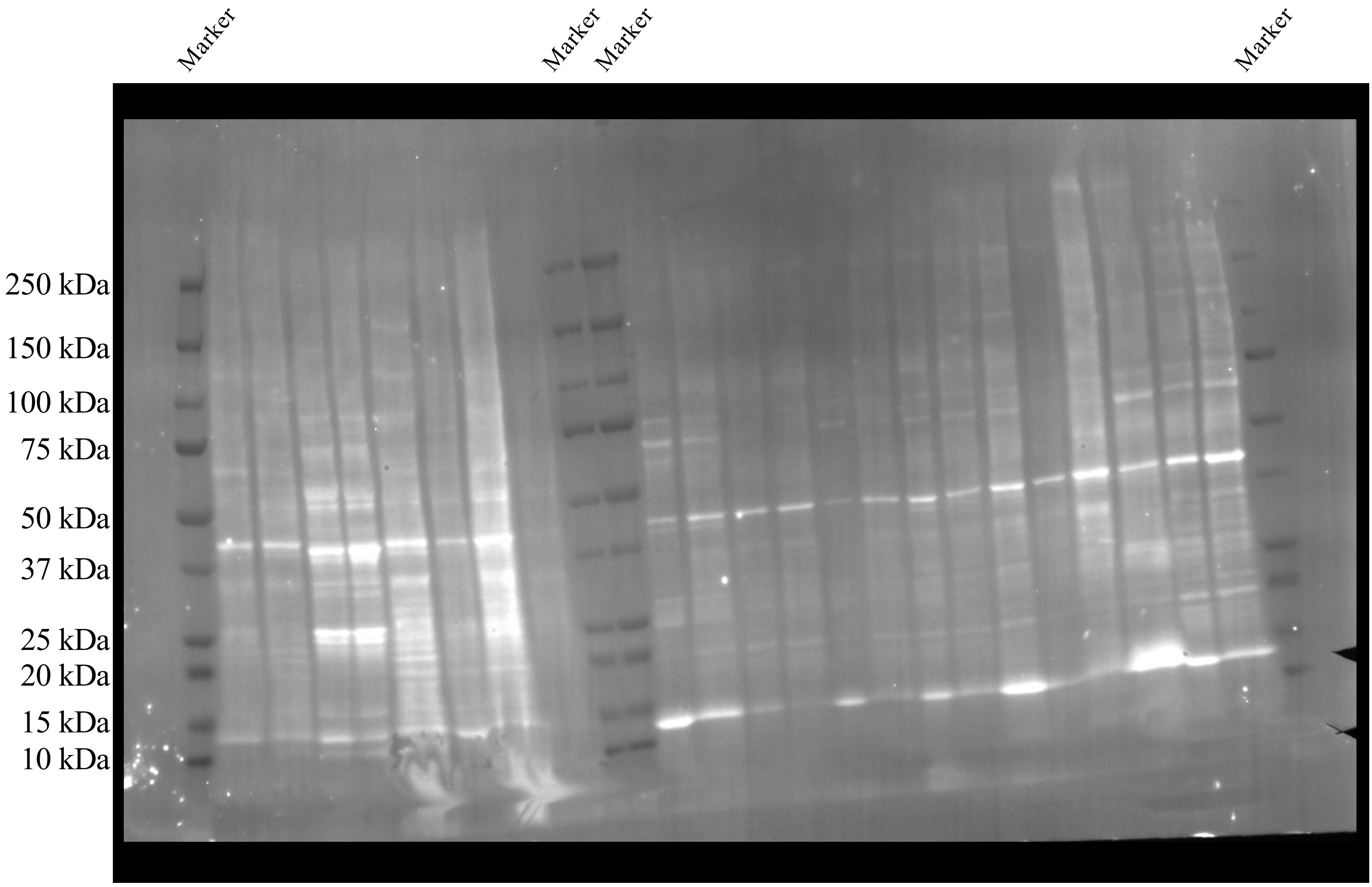

Uncropped images for Figure 1c and 1d - *middle cerebral artery*

Blot stained for TMEM16A

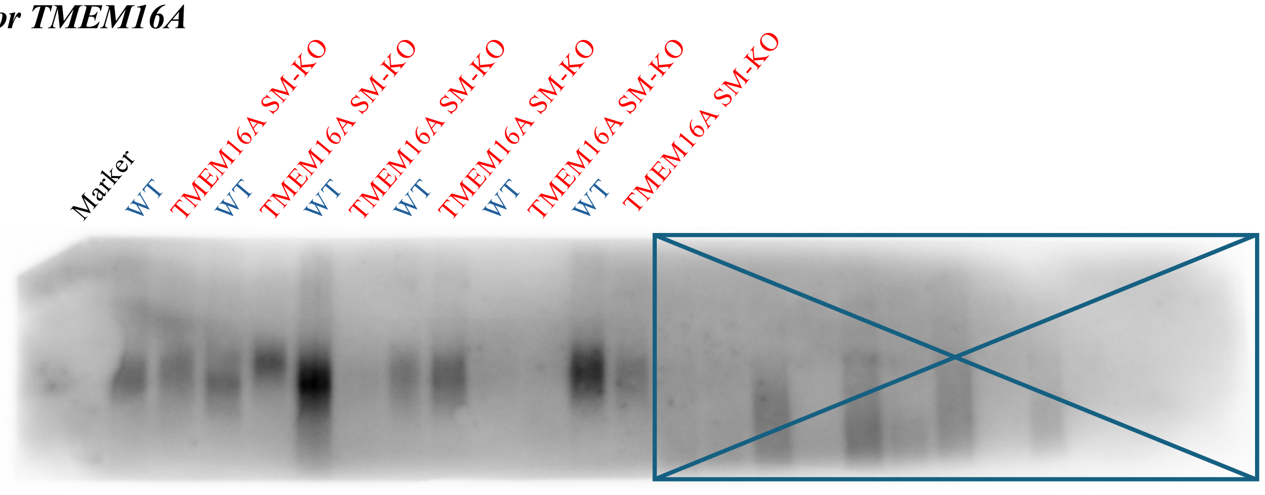

Corresponding molecular weight markers

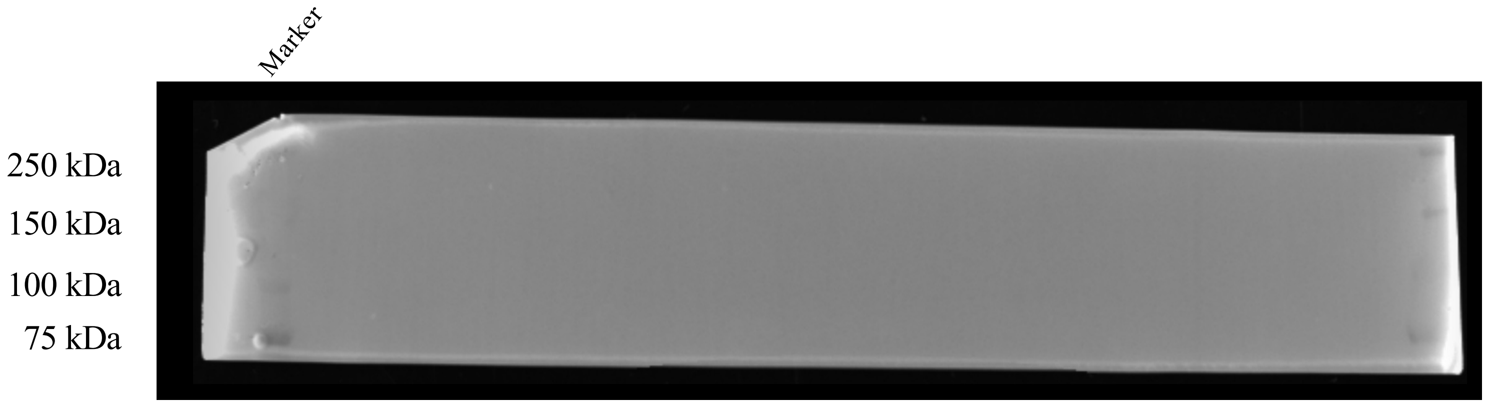

Membrane used for loading normalization

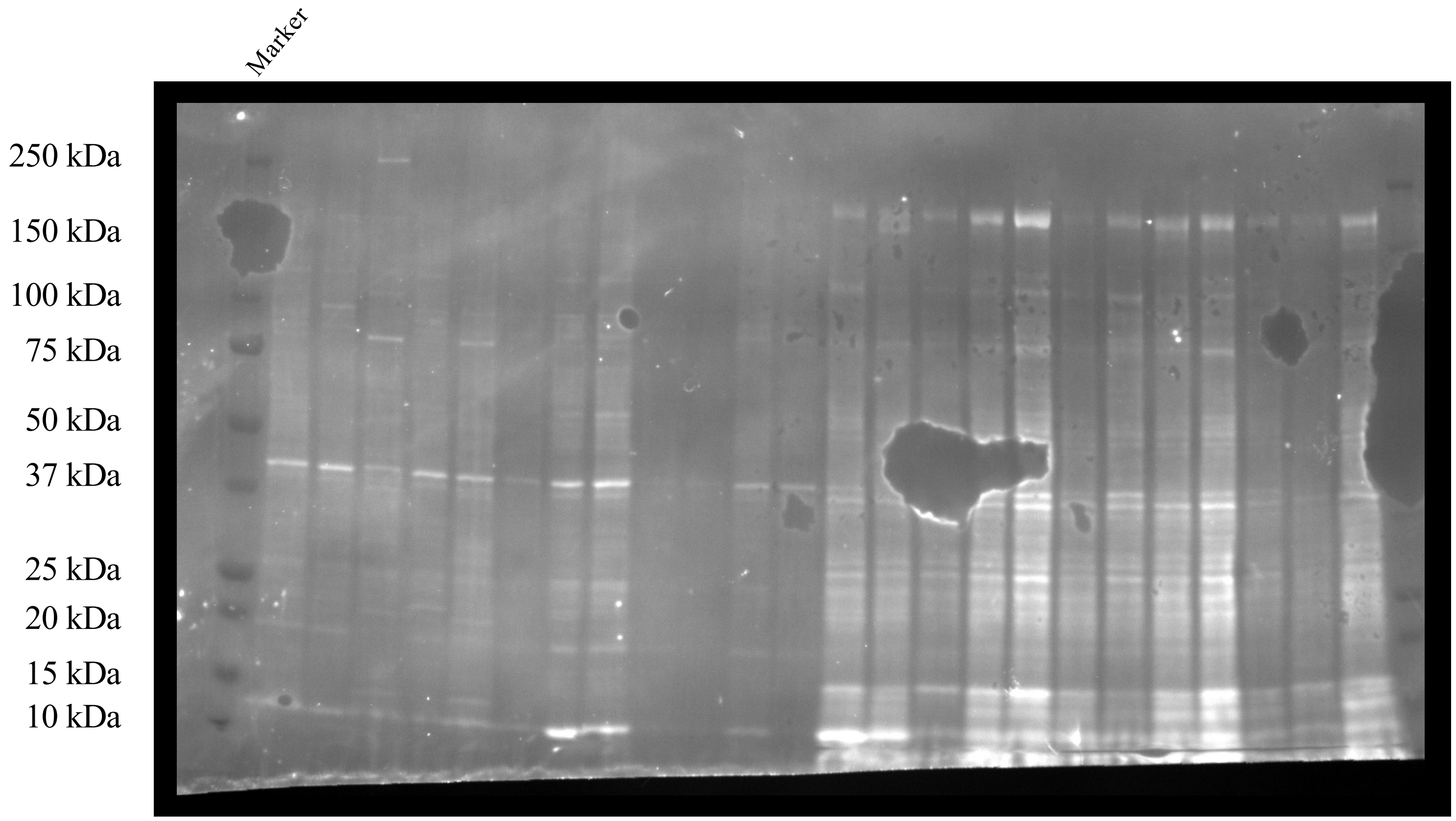

Uncropped images for Figure 1e and 1f - *heart lysate*

Blot stained for TMEM16A (upper), and blot stained for Pan-Actin (lower)

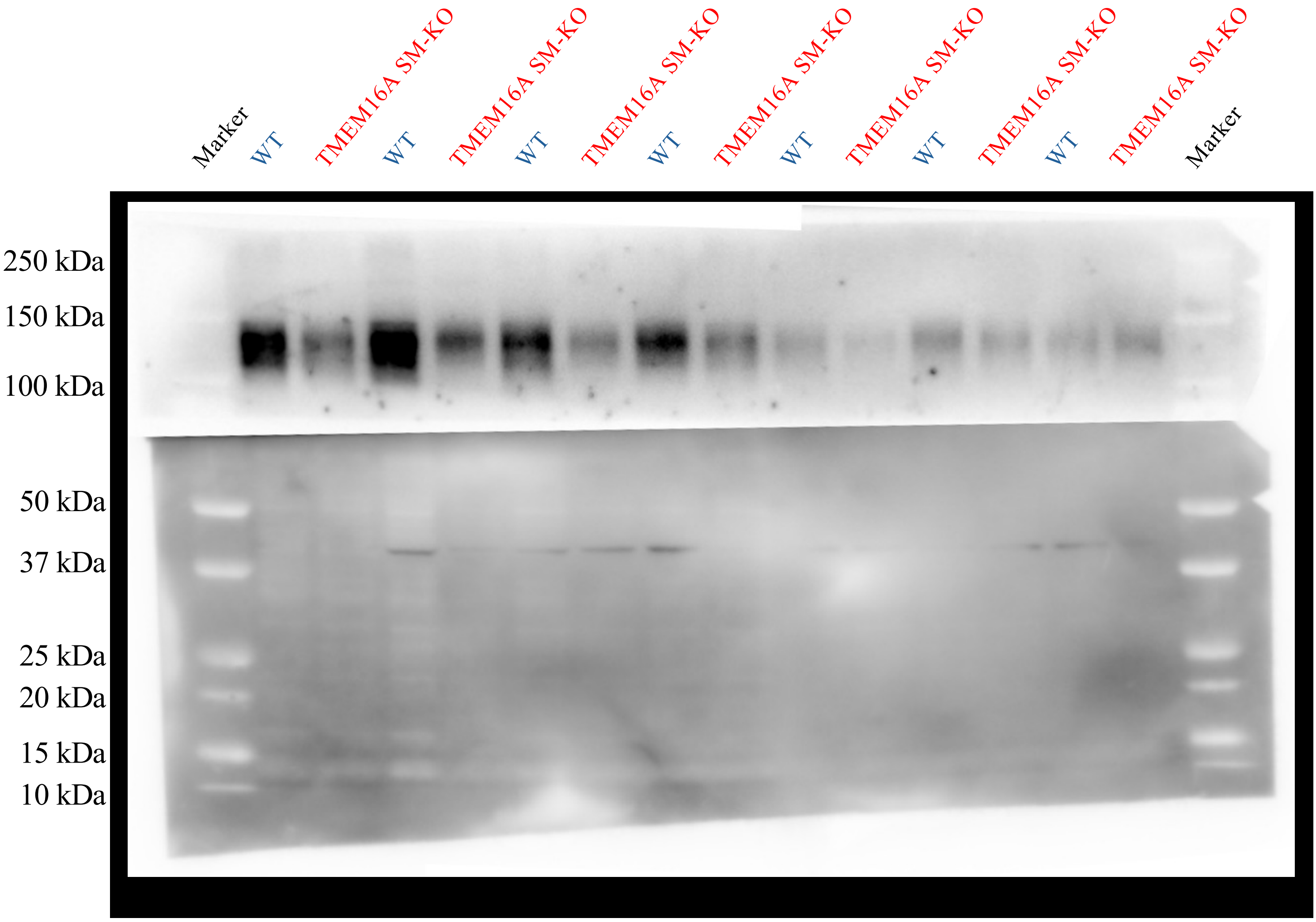

Supplement: Supplementary file 5 — Supplementary Material 5 (PDF 6.21 MB) [file 12975_2026_1471_MOESM5_ESM.pdf]
